# Genome-wide identification and characterization of *VQ* genes from cultivated peanut and their response to abiotic stresses

**DOI:** 10.3389/fpls.2026.1865834

**Published:** 2026-07-03

**Authors:** Yulong Liu, Rong Hu, Qianqian Wang, Shutao Yu, Haocui Miao, Chunjuan Li, Quanxi Sun, Shihua Shan, Cuiling Yuan

**Affiliations:** 1 Shandong Peanut Research Institute, Qingdao, China; 2 College of Life Sciences, Qingdao Agricultural University, Qingdao, China; 3 College of Agronomy, Qingdao Agricultural University, Qingdao, China; 4 Institute of Sandy Land Management and Utilization of Liaoning, Fuxin, China; 5 Crop Research Institute, Xinjiang Academy of Agricultural Sciences, Urumqi, China

**Keywords:** abiotic stresses, *AhVQ* gene family, expression patterns, genome-wide identification, peanut

## Abstract

Valine-glutamine (VQ) motif-containing proteins serve as pivotal regulators in plant growth, development, and abiotic stress responses. However, systematic genome-wide characterization of the *AhVQ* gene family in cultivated peanut (*Arachis hypogaea* L.) remains unreported. In this study, we identified 71 *AhVQ* genes unevenly distributed across 20 chromosomes; these genes were phylogenetically clustered into seven groups with *Arabidopsis* and rice VQs. Members of the same group displayed highly conserved exon-intron structures and protein motifs, and their promoters were enriched with abundant phytohormone-responsive and stress-related cis-elements, including ABRE, LTR, and CGTCA-motif. Tissue expression profiling revealed that 15 *AhVQ* genes (e.g., *AhVQ6*, *AhVQ8*, *AhVQ31*, and *AhVQ40*) exhibited constitutive expression in all 22 tested tissues, while other *AhVQ* genes showed tissue-preferential patterns: *AhVQ9* and *AhVQ17* in roots, *AhVQ65* in reproductive shoot tips, and *AhVQ38* in pistils. Transcriptome analyses demonstrated that all 8 differentially expressed *AhVQ* genes were upregulated in roots under drought stress, 20 of 21 *AhVQ* genes were induced under salt stress, and only 5 genes responded to cold stress in leaves (*AhVQ31* and *AhVQ67* were upregulated and *AhVQ33, AhVQ45*, and *AhVQ69* were downregulated). In silico prediction indicated extensive interactions between AhVQ proteins and WRKY transcription factors involved in stress signaling pathways. Collectively, our results provide comprehensive insights into the evolutionary characteristics, expression patterns, and stress response profiles of the peanut *VQ* gene family, offering key candidate genes for the genetic improvement of abiotic stress tolerance in peanut breeding.

## Introduction

Valine-glutamine (VQ) proteins constitute an ancient family characterized by a highly conserved FxxhVQxhTG motif, where “x” represents any amino acid and “h” denotes a hydrophobic amino acid. VQ proteins are categorized based on the terminal three amino acids of this conserved motif. According to differences in these amino acids, the VQ motif is classified into distinct types, such as LTG, FTG, VTG, and ITG (Liu et al., 2020). VQ proteins have a highly conserved VQ motif. Their amino acid sequences at other regions vary obviously. This variation results in functional differentiation among *VQ* family members (Jing and Lin, 2015).

The *VQ* gene family has been identified in a wide range of species (Tian et al., 2023a; Zhang et al., 2025). VQ motif-containing proteins were first identified in *Arabidopsis* (Cheng et al., 2012). Although VQ proteins were initially thought to be transcriptional regulators exclusive to plants, recent studies have detected them in fungi, indicating that the *VQ* gene family is widely distributed (Jiang et al., 2018). Currently, 29, 39, 113, and 61 VQ-related proteins have been identified in *Arabidopsis*, rice, wheat, and maize, respectively (Cheng et al., 2012; Kim et al., 2013; Song et al., 2015; Zhang et al., 2022a). Conserved motif analysis detected six VQ motif types in *Arabidopsis* and four in rice. Phylogenetic analysis classified VQ proteins from the two species into seven subgroups (I–VII). Subgroups V and VI contain FTG and VTG/ITG-type motifs, and the rest belong to the LTG type (Cheng et al., 2012; Kim et al., 2013). Gene structure analysis has shown that most *VQ* genes lack introns (Liu et al., 2020).

Recently, several studies have reported that *VQ* genes play important roles in abiotic stress responses (Li et al., 2014; Wang et al., 2015; Zhong et al., 2021; Hao et al., 2022). For instance, *CmVQ24* and *CmVQ4–2* in *Cucumis melo* participate in the ABA signaling pathway to enhance plant adaptation to abiotic stresses (Zhang and Wei, 2019). *IbVQ4* functions in conjunction with *IbWRKY2* to improve the response capacity of sweet potato to drought and salt stress (Zhu et al., 2020). *ZmVQ19* is strongly induced by NaCl and drought treatment, and its mutants exhibited improved seedling growth under these conditions (Song et al., 2015). *AtVQ9* is highly expressed in roots and responds strongly to salinity stress (Hu et al., 2013). Transgenic *Arabidopsis* plants overexpressing *StVQ31* show enhanced salt tolerance by maintaining osmosis and antioxidant homeostasis of plant cells under high salt stress (Zhai et al., 2024). Similarly, overexpression of *SlVQ16* reduces salt sensitivity in tomatoes. This finding demonstrated that *SlVQ16* acts as a positive regulator of salt stress in tomato (Ma et al., 2023).

VQ proteins in plants interact with transcription factors (TFs), such as WRKY, through a unique conserved domain and play an important role in WRKY-mediated signaling pathways (Dong et al., 2003; Song et al., 2016; Wang et al., 2019b; Yuan et al., 2020). Thus, VQ proteins can influence the transcriptional activity of TFs and are involved in plant development, stress responses, and hormone signaling pathways (Cheng et al., 2012; Wang et al., 2015; Zhou et al., 2016). For example, *RsVQ4* interacts with *RsWRKY26* to positively regulate the heat stress response in radish (He et al., 2023). *SlVQ10* physically interacts with *SlWRKY51* to enhance chilling tolerance in tomato by promoting proline accumulation (Wang et al., 2024). *OsVQ8* positively regulates thermotolerance by modulating reactive oxygen species (ROS) balance and the hypersensitive response. The balance between destructive and protective responses in *OsVQ8*-overexpressing plants is more stable and less susceptible to disruption by heat stress compared to that in wild-type plants (Chen et al., 2022).

Peanut (*Arachis hypogaea* L.), an allotetraploid legume originating from the tropical and subtropical regions of South America, is one of the most important oilseed and economic crops worldwide. With increasing challenges from abiotic stresses such as drought, salinity, and low temperature, improving stress tolerance has become a key objective to ensure peanut yield and quality (Xu et al., 2021; Gundaraniya et al., 2023; de Camargo Santos et al., 2024). VQ motif-containing proteins function as essential regulators in plant growth, development, and stress responses. However, the *AhVQ* gene family has not yet been systematically identified or functionally characterized in peanut. As an allotetraploid crop, peanut possesses a large number of homeologous gene pairs. Whether *AhVQ* homeologs have functional divergence and biased expression is still unclear, which forms an important research gap. In this study, we present the genome-wide analysis of the *VQ* gene family in peanut, identifying 71 *AhVQ* genes that cluster into seven distinct subfamilies. We systematically investigated their evolutionary relationships, gene structures, cis-regulatory elements, and protein interaction networks, and profiled their expression patterns across developmental stages and in response to major abiotic stresses. Our findings establish a foundation for understanding the roles of *AhVQ* genes in stress signaling and provide valuable genetic resources for the molecular breeding of stress-tolerant peanut varieties.

## Materials and methods

### Identification of the *VQ* family from *A. hypogaea*

The peanut genome sequence, CDS, and protein sequences based on the reference genome assembly Tifrunner.gnm2.J5K5 (BioProject: PRJNA419393) were downloaded from the PeanutBase (https://www.peanutbase.org/). Each AhVQ protein sequence was aligned using the NCBI Conserved Domain Search (https://www.ncbi.nlm.nih.gov/Structure/bwrpsb/bwrpsb.cgi) to determine the position of the VQ motif. Sequences lacking the VQ domain were manually excluded to finalize the identification of *AhVQ* genes. The biophysical properties of AhVQ proteins, including peptide length (aa), isoelectric point (pI), and molecular weight (MW), were estimated using the online tool ProtParam (https://web.expasy.org/protparam/) (Gasteiger et al., 2003).

### Multiple sequence alignment and phylogenetic analysis

Twenty-nine *Arabidopsis* and 39 rice VQ protein sequences were downloaded from the TAIR (https://www.arabidopsis.org/) and RGAP (https://rice.uga.edu/) databases, respectively. Multiple sequence alignments were performed using the ClustalW tool for VQ proteins from *Arabidopsis*, rice, and peanut. Based on the alignment results, a phylogenetic tree was constructed using MEGA 11 software through the neighbor-joining (NJ) method. The Poisson substitution model was adopted, and pairwise deletion was used for missing data treatment, with 1000 bootstrap replicates to assess tree reliability (Kumar et al., 2016).

### Chromosomal position of the conserved motif and gene structure construction

The physical locations of *AhVQ* genes were visualized and analyzed using the online tool MG2C (http://mg2c.iask.in/mg2c_v2.1/). Conserved motifs of *AhVQ* genes were identified using MEME (https://meme-suite.org/meme/), with the maximum number of motifs set to 10 (Bailey et al., 2009). The conserved motifs were plotted using TBtools (Chen et al., 2020). According to the CDS and related genome sequences of *AhVQ* genes, the structure of these genes were mapped using the online website GSDS (https://gsds.gao-lab.org/) (Hu et al., 2015).

### Prediction of the AhVQ protein interaction network

The protein interaction network was predicted using the STRING database (https://string-db.org) (Szklarczyk et al., 2021). Protein sequences of AhVQ and AhWRKY TFs were mapped to the *Arabidopsis* database by constructing an *Arabidopsis* association model.

### Cis-elements in the promoter regions of *AhVQ* genes

The upstream regulatory regions of *AhVQ* genes (2,000 bp upstream of the start codon ATG) were retrieved from the peanut genome. These sequences were used to predict potential cis-acting elements using PlantCARE (https://bioinformatics.psb.ugent.be/webtools/plantcare/html/) (Lescot et al., 2002).

### Peanut materials and growth conditions

The cold-tolerant peanut cultivar Huayu 6316 was used in this study. Seeds were sown evenly in Petri dishes and watered with distilled water to promote germination. Seedlings were cultured in an artificial climate chamber maintained at 20°C with a 16/8-h photoperiod (day/night). At the three-leaf stage, seedlings were subjected to three distinct abiotic stress treatments: 20% PEG-6000 (polyethylene glycol 6000) for drought stress, 1% NaCl for salt stress, and 4°C for cold stress (Liu et al., 2019; Wang et al., 2022; Zhang et al., 2022b). Plant material was sampled at 2 h after treatment, with three biological replicates for each treatment condition. The second compound leaves were collected for the cold stress treatment, whereas roots were collected for the drought and salt treatments. All samples were frozen in liquid nitrogen and stored at -80°C.

### Transcriptome sequencing and analysis

The samples were sent to Shanghai OE Biotech Co., Ltd. for RNA extraction. Quality-confirmed RNA was subsequently used for library construction and transcriptomic sequencing. Raw sequencing data were filtered using fastp software to obtain clean reads, followed by systematic quality assessment. Differentially expressed genes (DEGs) between samples were identified using the DESeq2 package in R. Two criteria were adopted for pairwise expression comparison of each gene based on RNA-seq data: expression fold change and statistical significance. The expression fold change between the treatment and control groups was calculated as log_2_FC = log_2_(Fold Change). The initial *P*-value of each gene was calculated, and the false discovery rate (FDR) method was applied to conduct multiple hypothesis testing correction to obtain the q-value (adjusted *P*-value). By default, genes with |log_2_FC| ≥ 1 and q-value < 0.05 were defined as DEGs. When the number of screened DEGs was insufficient, the threshold was adjusted to |log_2_FC| ≥ 1 and *P*-value < 0.05 for alternative screening. DESeq was used by default for differential expression analysis when biological replicates were unavailable. Subsequently, GO functional annotation and KEGG pathway enrichment analyses of DEGs were performed using the online OECloud tools (https://cloud.oebiotech.com). All statistical data were organized and visualized using Microsoft Excel 2019 and GraphPad Prism 9.

### Tissue expression analysis of *AhVQ* genes

To investigate the tissue-specific expression patterns of *AhVQ* genes, RNA-seq data for peanut developmental time-course tissues were retrieved from the NCBI database under accession number PRJNA291488 (Clevenger et al., 2016). A total of 22 different peanut tissue samples covering continuous developmental stages were included in the subsequent expression analysis. Homologous gene matching was performed to screen and characterize all candidate sequences, and an expression heatmap of 71 *AhVQ* genes was constructed based on the normalized log_2_FPKM expression values using the online Metware Cloud platform (https://cloud.metware.cn).

### RNA isolation and quantitative RT-PCR

RNA was extracted from control and treated peanut samples using the TaKaRa Mini BEST Plant RNA Extraction Kit (TaKaRa Biotechnology Co., Ltd., Beijing, China). cDNA was prepared following the user manual of the PrimeScript™ FAST RT Reagent Kit with gDNA eraser (Takara Biomedical Technology, Ltd., Beijing, China). Primers for 10 representative *AhVQ* genes were designed using the Primer-BLAST tool. qRT-PCR was performed on an ABI7500 system (Applied Biosystems, Foster City, CA, USA) (Li et al., 2021). The reaction system comprised 10 μL of 2× TB Green Premix Ex Taq II Fast qPCR, 0.8 μL of 10 μmol/L forward primer, 0.8 μL of 10 μmol/L reverse primer, 0.4 μL of ROX Reference Dye II, 2 μL of cDNA, and 6 μL of RNase‐free water. The β-actin gene was employed as the internal reference gene, and the amplification efficiencies of the reference gene and all target genes ranged between 97% and 102% (Yuan et al., 2019). Primers used in this study were listed in **Supplementary Table 1**. The amplification program included an initial denaturation at 95°C for 30 s, followed by 40 cycles of 95°C for 5 s and 60°C for 34 s, and a final dissociation stage at 95°C for 15 s, 60°C for 1 min, and 95°C for 15 s. Three biological replicates and three technical replicates were performed for each treatment. The relative expression of genes was calculated using the 2^−ΔΔCt^ method (Livak and Schmittgen, 2001). Fluorescent quantitative primers were designed using Primer-BLAST (https://www.ncbi.nlm.nih.gov/tools/primer-blast/index.cgi). The raw sequencing data generated in this study have been deposited in the NCBI Sequence Read Archive under accession number PRJNA1335572. The peanut reference genome used in this study is Tifrunner.gnm2.J5K5.

## Results

### Chromosomal location and duplication analysis of *AhVQ* genes

**Figure 1:**
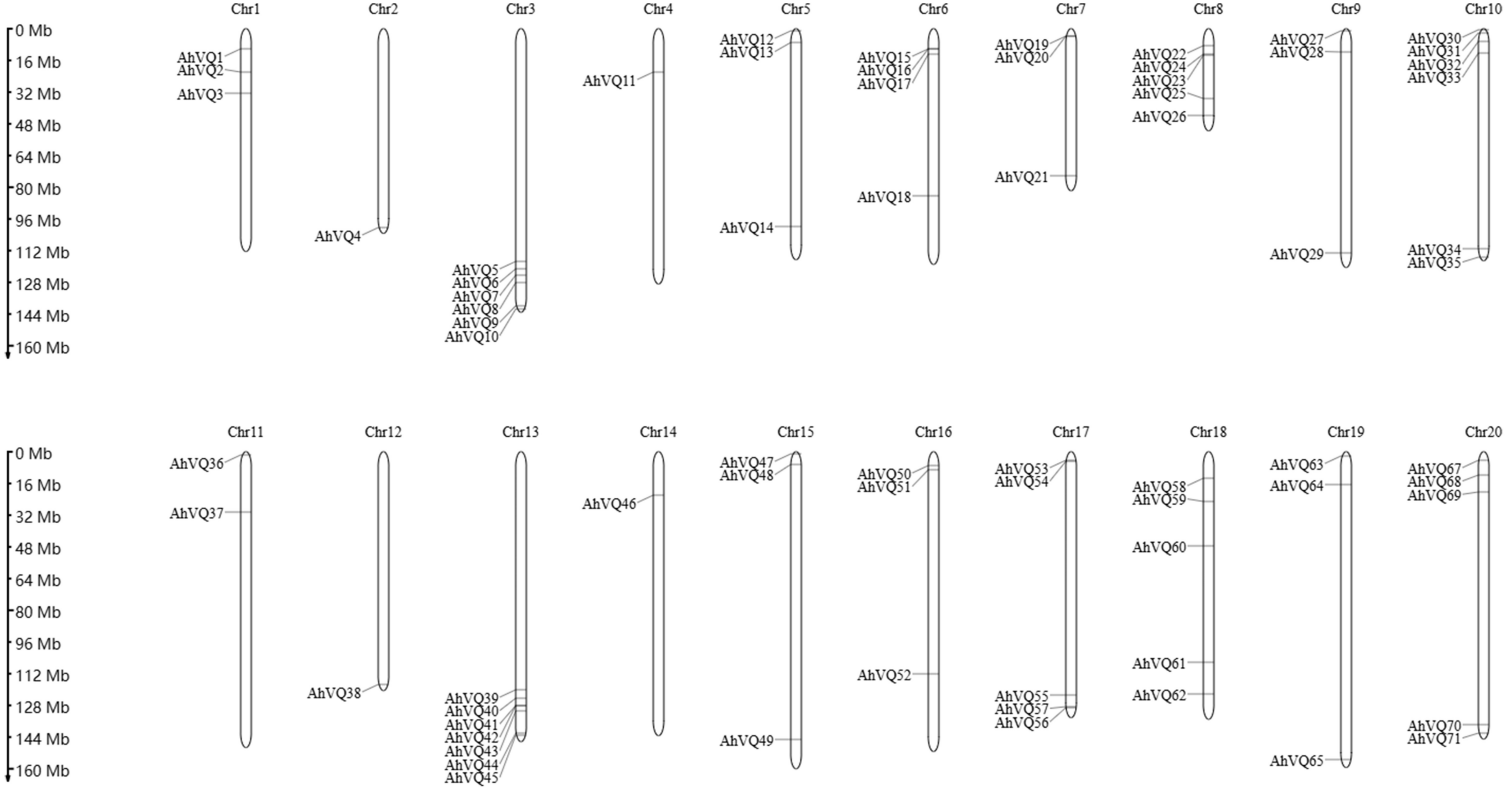
Chromosomal distribution of *AhVQ* genes. Chromosome numbers are listed above the chromosomes, while chromosome sizes are indicated in megabases (Mb) on the left side of the figure.

Genome-wide screening identified 71 non-redundant *AhVQ* genes in the cultivated peanut genome, sequentially designated *AhVQ1* to *AhVQ71* according to their physical positions on 20 chromosomes (**Figure 1**). The number of genes per chromosome ranged from 1 (chromosomes 2, 4, 12, and 14) to 7 (chromosome 13). Notably, most *AhVQ* genes were concentrated in the terminal regions of chromosomes, and certain genomic regions with a high density of *AhVQ* genes were observed on specific chromosomes, such as chromosomes 3 and 13. This distribution suggests the existence of *AhVQ* gene-enriched regions within the peanut genome. Protein sequence alignment revealed that all AhVQ proteins contained the conserved motif FxxhVQxhTG. Furthermore, the main physicochemical properties of the 71 AhVQ proteins were analyzed, including amino acid length, molecular weight (MW), and theoretical isoelectric point (pI). The lengths of all AhVQ proteins ranged from 79 to 489 amino acids (aa), with an average of 208.13 aa. The MW of these AhVQ proteins varied from 53.25 to 98.42 kDa, and pI values ranged from 4.37 to 11.66. Detailed information regarding all *AhVQ* genes is provided in **Supplementary Table 2**.

**Figure 2:**
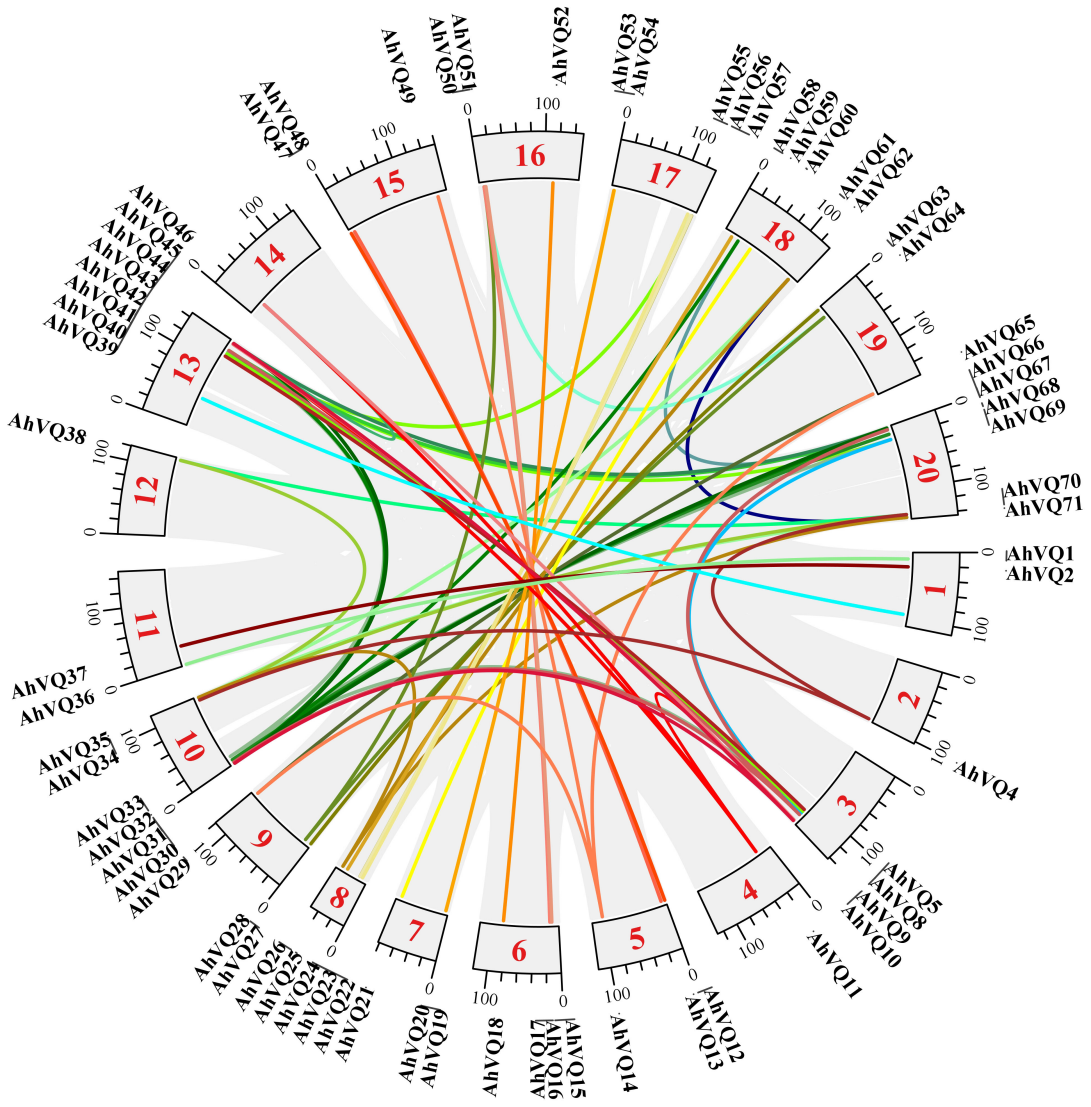
Duplication and synteny of *AhVQ* genes. Gray lines indicate whole-genome duplication blocks, and colored lines indicate segmental duplication pairs of *AhVQ* genes.

No significant positive correlation was detected between genome size and the size of the *AhVQ* gene family. Segmental duplication and tandem duplication were the drivers for the expansion of *AhVQ* genes in peanut. A total of 61 segmental duplication pairs covering all 71 *AhVQ* genes and two tandem duplication pairs (*AhVQ15*/*AhVQ16*, *AhVQ19*/*AhVQ20*) were identified (**Figure 2**). Notably, several *AhVQ* genes were involved in more than one duplication event. These results indicate that both tandem duplication and segmental duplication collectively contributed to the expansion of the *AhVQ* gene family. This event arises from frequent unequal crossing-over in local chromosomal regions during peanut evolution. It is a key mechanism underlying the rapid expansion of gene families in plant genomes. The abundant paralogous genes generated by tandem duplication can gradually undergo functional divergence through sequence variation, thereby enhancing the functional diversity of the gene family. Additionally, these duplications may increase gene expression levels and improve plant tolerance to environmental stresses. Notably, *AhVQ4*, *AhVQ31*, and *AhVQ65* were located on different chromosomes and covered by segmental duplication events, indicating that these key genes were conserved during peanut genome evolution and may perform essential biological functions.

### Phylogenetic, conserved motif, and gene structure analysis

**Figure 3:**
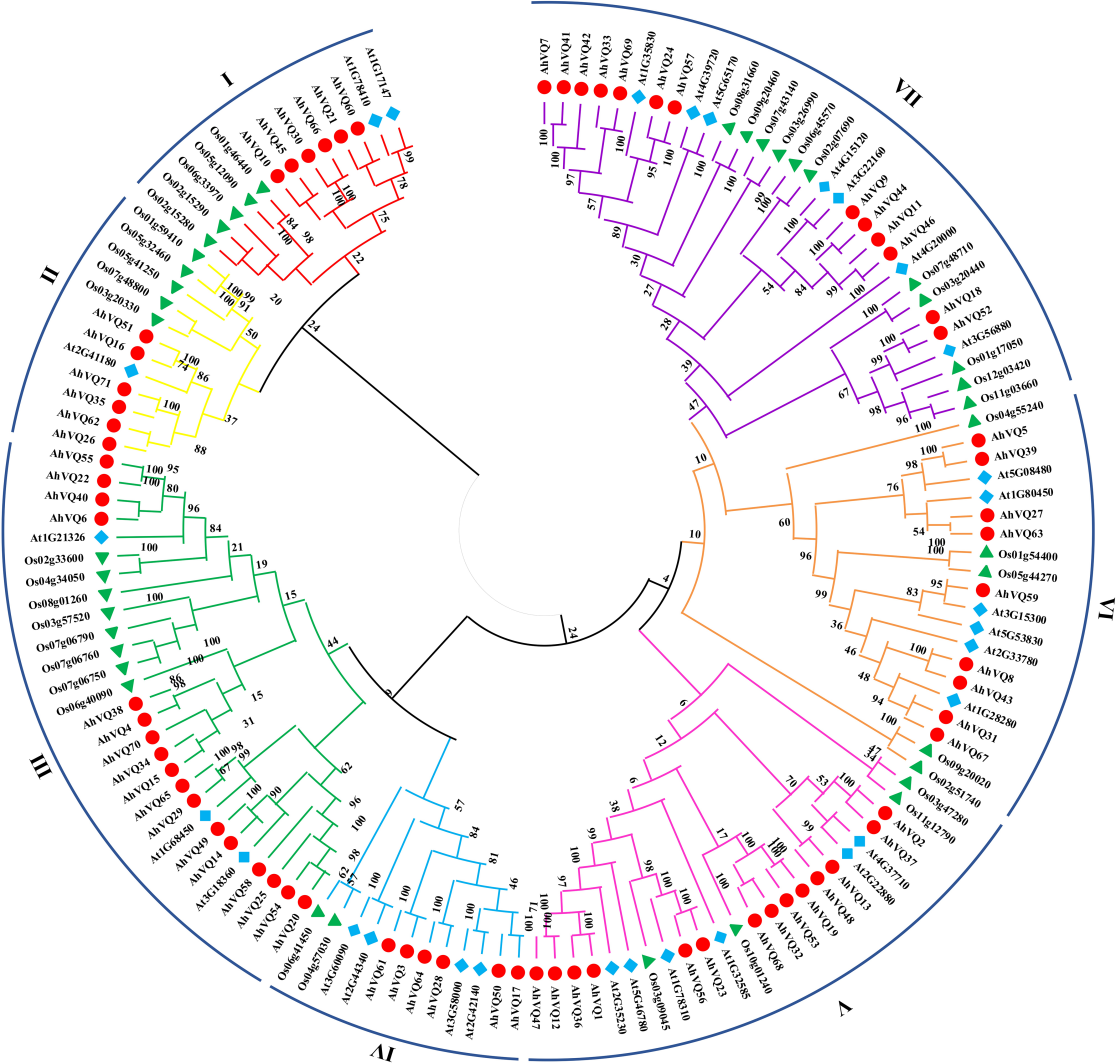
Phylogenetic tree of the *VQ* family from different species. The phylogenetic tree of VQ proteins from peanut, rice, and *Arabidopsis* was constructed using the neighbor-joining method in MEGA 11. The 71 AhVQ proteins, 29 *Arabidopsis* VQ proteins, and 39 rice VQ proteins were clustered into seven subgroups. Proteins from peanut, rice, and *Arabidopsis* are indicated by red circles, blue squares, and green triangles, respectively.

To elucidate the phylogenetic relationships of VQ proteins, a phylogenetic tree was constructed using MEGA 11 based on 139 VQ proteins from peanut, *Arabidopsis*, and rice. Phylogenetic analysis clustered the 71 AhVQ proteins with 29 AtVQs and 39 OsVQs into seven distinct subfamilies (I-VII). Subgroups I and II contained the fewest members (6 each), while Subgroup III was the largest with 17 members (**Figure 3**). Multiple sequence alignment of AhVQ proteins (**Supplementary Figure 1**) revealed that subgroup VI belonged to the FTG and VTG types, subgroup V belonged to the ITG type, and the remaining subgroups belonged to the LTG type. This classification is consistent with previous findings in *Arabidopsis* and rice (Kim et al., 2013). Additionally, although *AhVQ4*, *AhVQ15*, *AhVQ31*, and *AhVQ65* were distributed across different subgroups, all of them harbored the complete and conserved VQ motif, implying their functional conservation and potential roles in stress responses.

To gain insights into the functional regions of AhVQ proteins, the MEME (Multiple Em for Motif Elicitation) program was employed to identify conserved motifs among the 71 AhVQ proteins. A total of 10 conserved motifs (Motifs 1–10) were identified. These conserved motifs were represented by colored boxes scaled according to their width, which ranged from 6 to 21 aa residues. Among these, Motif 1 encoded the conserved VQ domain (**Supplementary Table 3**). All AhVQ proteins harbored the conserved VQ motif, and members within the same phylogenetic group generally possessed multiple identical or similar conserved motifs, exhibiting a highly consistent motif composition pattern.

**Figure 4:**
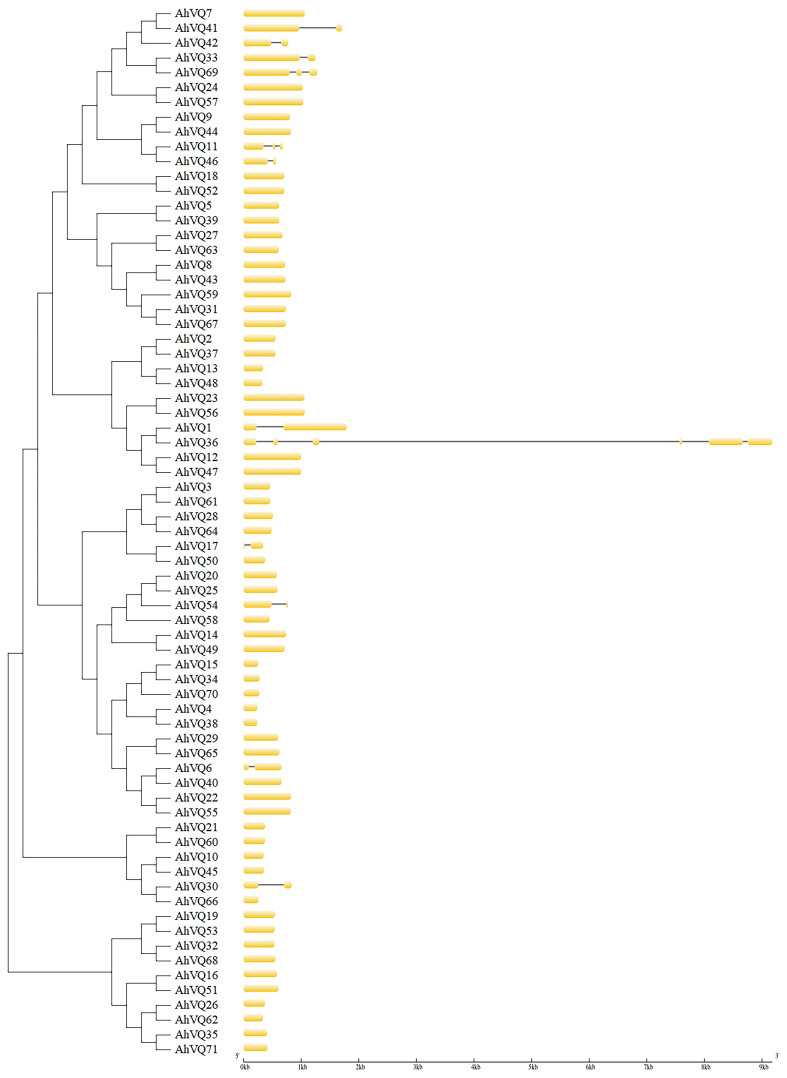
Gene structure of *AhVQ* genes. Exon-intron structure analysis of the *VQ* genes from peanut; yellow boxes and black lines represent exons and introns, respectively. Box and line lengths are displayed proportionally to gene length.

GSDS was used to analyze the exon-intron distribution of *AhVQ* genes, thereby clarifying their structural characteristics. As shown in **Figure 4**, 59 of the 71 (83.1%) *AhVQ* genes lacked introns, while the remaining 12 harbored at least one intron, with intron sizes varying according to the lengths of the respective genes. *AhVQ* genes within the same subgroup exhibited consistent structural patterns. For example, *AhVQ4*, *AhVQ34*, and *AhVQ65*, which are members of subgroup III, all featured an intron-free structure, whereas *AhVQ41* and *AhVQ42* in subgroup VII each contained a single intron.

### Cis-element analysis of the *AhVQ* gene promoter region

**Figure 5:**
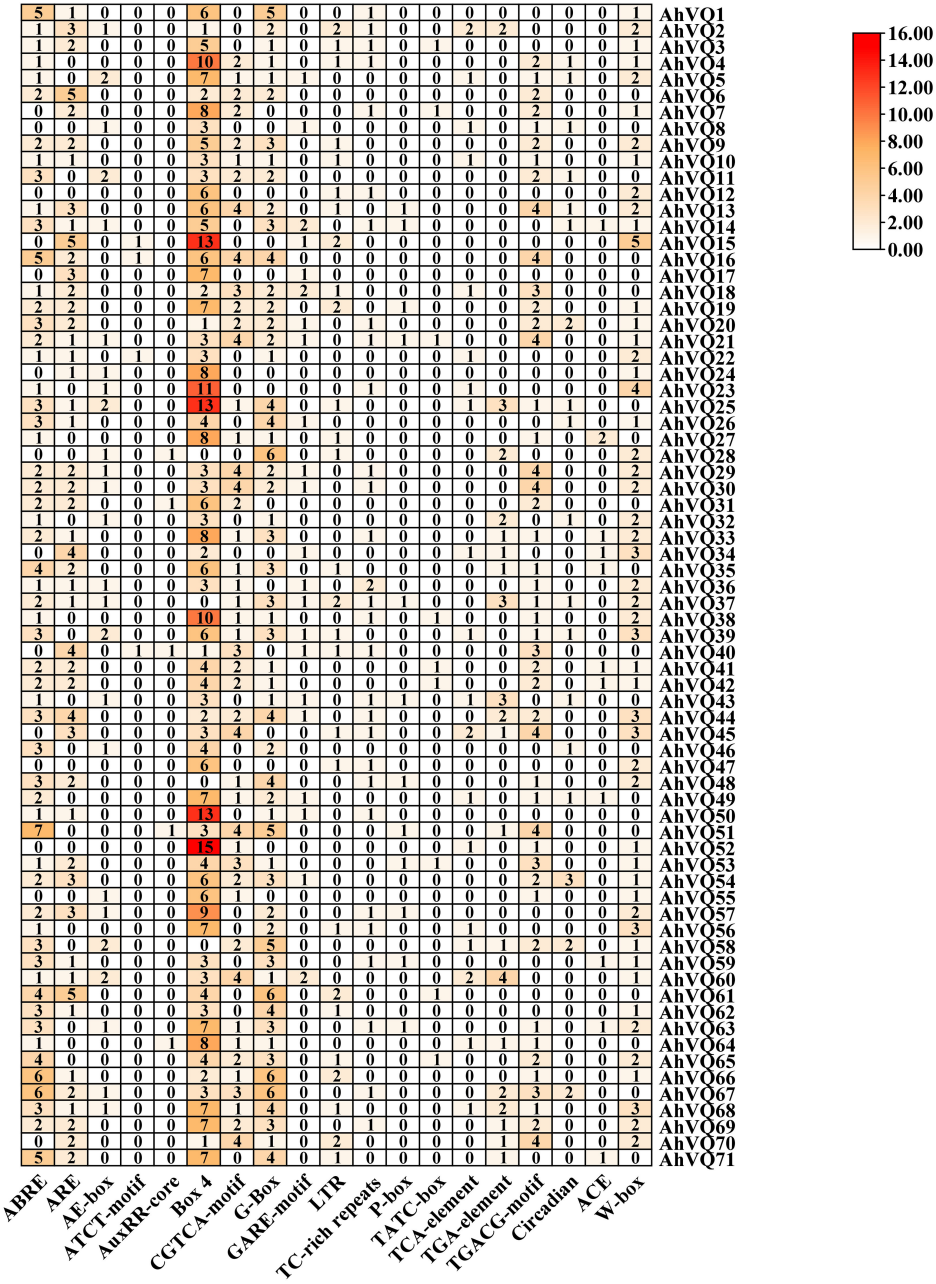
Analysis of cis-acting elements in the promoters of *AhVQ* genes. The abscissa represents the names of cis-acting elements, and the ordinate denotes the names of *AhVQ* genes. The color intensity in the plot indicates the number of elements, with the color gradient from white to red corresponding to an increase in the number of elements.

To explore the potential functions of *AhVQ* genes, cis-acting elements in their promoter regions were predicted and analyzed. Using PlantCARE, a total of 18 cis-acting elements were identified in the *AhVQ* promoter regions (2000 bp upstream of the translation start site). These elements were categorized into two groups: phytohormone-responsive elements and stress-related responsive elements. The hormone-responsive elements included one abscisic acid-responsive element (ABRE), two auxin-responsive elements (AuxRR-core and TGA-element), two MeJA-responsive elements (CGTCA-motif and TGACG-motif), three gibberellin-responsive elements (P-box, TATC-box, and GARE-motif), and one salicylic acid-responsive element (TCA-element). The nine stress-related elements included ARE, AE-box, ATCT-motif, Box 4, G-Box, LTR, TC-rich repeats, circadian, and ACE. A total of nine hormone-responsive cis-elements were identified. ABRE motifs were widely distributed in AhVQ promoter regions and present in 83.67% of *AhVQ* genes. Two MeJA-responsive elements, CGTCA-motif and TGACG-motif, shared the same occurrence frequency of 67.35%. As shown in **Figure 5**, the promoters of *AhVQ4*, *AhVQ10*, *AhVQ15*, *AhVQ31* and *AhVQ65* were enriched in stress-related cis-elements such as ABRE, LTR, TC-rich repeats, and W-box. Among the 71 identified AhVQ proteins, 51 harbored at least one W-box element. Several members, including AhVQ15, AhVQ23, AhVQ34, and AhVQ45, contained 3 to 5 W-box motifs. Although these elements were not present in all genes, their high overall frequency of distribution suggests that these *AhVQ* members may play important regulatory roles in plant responses to abiotic stresses by participating in multiple signaling pathways. Additionally, the widespread occurrence of W-box elements in stress-responsive AhVQ promoters implies potential regulatory interactions between AhVQ proteins and WRKY TFs, laying a preliminary foundation for the subsequent protein-protein interaction prediction.

### Expression patterns of *AhVQ* genes in different tissues

GO and KEGG enrichment analyses indicated that *AhVQ* genes are functionally diverse and are involved in transcriptional regulation, hormone signaling, stress responses, and circadian rhythm, with significant differential expression among family members. This extensive enrichment highlights the functional diversity of *AhVQ* genes and their distinct regulatory roles. The divergent enrichment patterns among family members also suggest evident differential expression, providing a basis for further analysis of their expression profiles.

**Figure 6:**
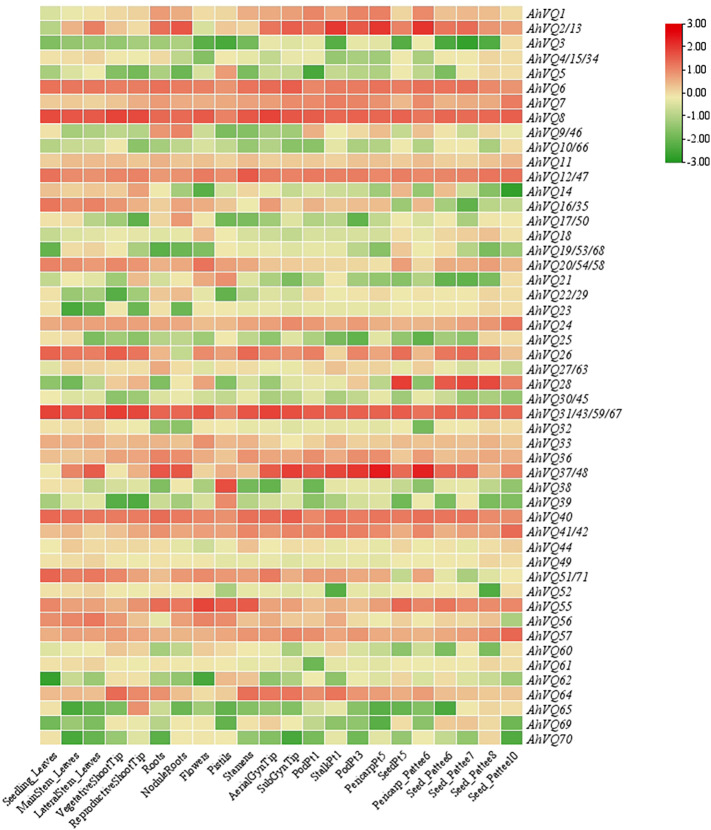
Expression of *AhVQ* genes in different tissues of peanut at various developmental stages. The heatmap was generated using TBtools, with expression levels shown as log_2_-transformed values. The tissues used for expression profiling are indicated at the bottom of each column. The orthologous genes in wild peanut are provided to the right of the heatmap. The color gradient (red/black/green) indicates gene expression levels from high to low.

To clarify the expression patterns and potential functions of *AhVQ* genes in different peanut tissues, transcriptome data of 22 distinct peanut tissues, including leaves, shoot apices, roots, root nodules, perianths, flowers, pistils, stamens, peg tips, pods, pericarps, and seeds, at various developmental stages were acquired from public databases. Analysis based on log_2_FPKM values revealed pronounced expression variations of *AhVQ* genes among these tissues (**Figure 6**). Correlation analysis of tissue expression profiles and phylogenetic classification indicated that genes belonging to the same subfamily shared similar expression characteristics, which reflected functionally conservative properties within each subgroup. Several genes, such as *AhVQ6*, *AhVQ8*, *AhVQ31*, and *AhVQ40*, exhibited constitutive expression across all tested tissues, whereas other members displayed apparent tissue-specific expression patterns. Specifically, Subgroup VI genes *AhVQ31* and *AhVQ67* maintained widespread high expression and served as housekeeping genes regulating peanut growth and development. Subfamily II genes *AhVQ4* and *AhVQ9* accumulated predominantly in roots, possibly participating in root growth and underground stress response. Subgroup III members displayed divergent expression profiles: *AhVQ15* and *AhVQ34* were highly expressed in roots, *AhVQ65* was specifically detected in reproductive shoot tips, *AhVQ17* was restricted to root tissues, and *AhVQ38* was uniquely expressed in pistils, demonstrating functional differentiation of paralogous genes within the same subfamily. Collectively, 15 *AhVQ* genes from diverse subfamilies sustained persistent high expression and exerted essential effects on peanut growth and development.

### Expression patterns of *AhVQ* genes under different stresses

**Figure 7:**
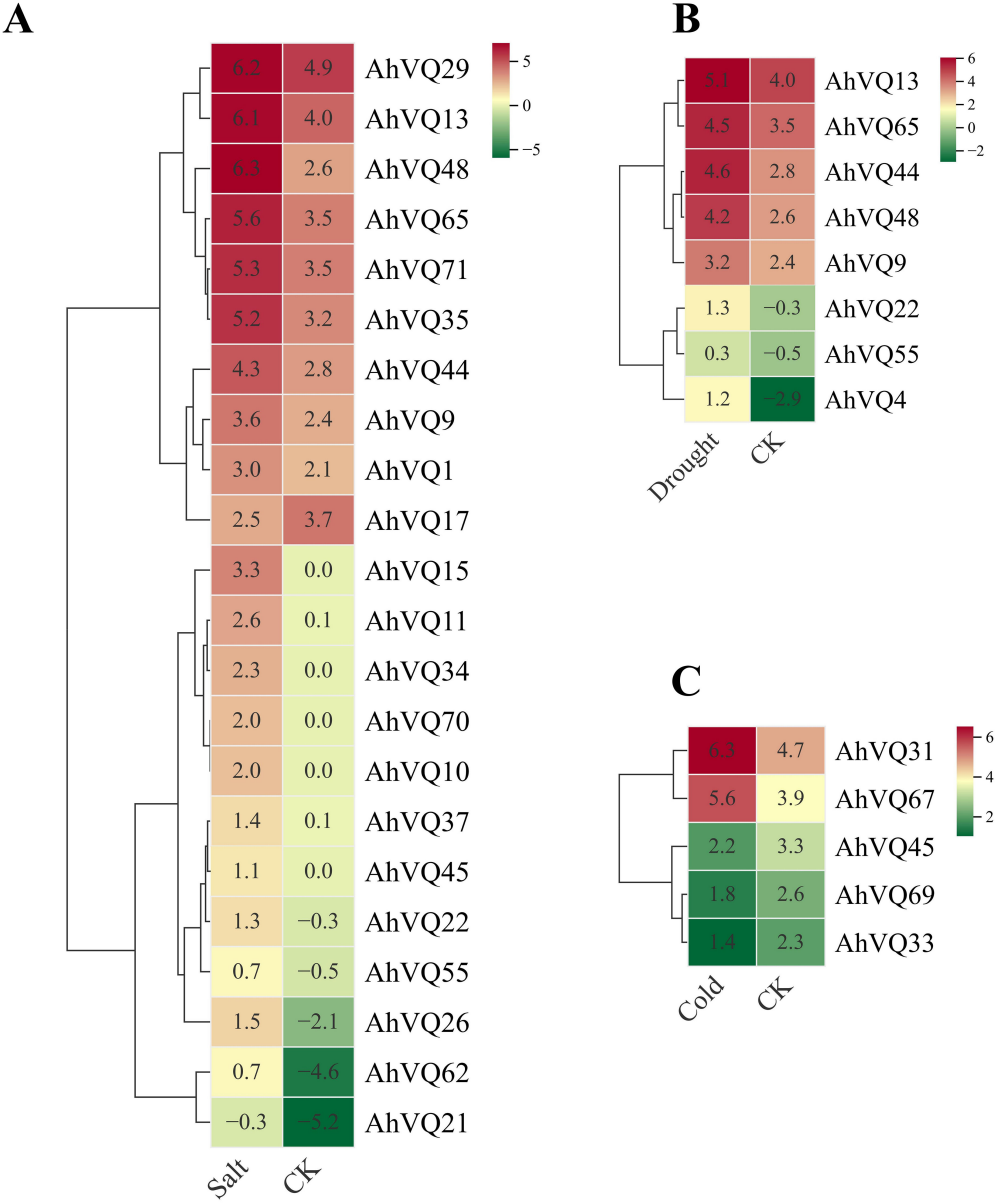
Expression analysis of *AhVQ* genes under different stresses. The heatmap was generated using HemI software based on log_2_-transformed fragments per kilobase of transcript per million (FPKM) values of *AhVQ* genes. Red and green colors represent high and low expression levels, respectively. **(A)** Differential expression patterns of *AhVQ* genes under salt stress (1% NaCl). **(B)** Differential expression patterns of *AhVQ* genes under drought stress (20% PEG-6000). **(C)** Differential expression patterns of *AhVQ* genes under cold stress (4 °C). CK indicates the expression levels of different *AhVQ* genes under control conditions without any treatment.

DEGs under various stress conditions were screened using thresholds of ∣log_2_FC∣≥ 1 and q-value The expression patterns of *AhVQ* genes were analyzed using RNA-seq data. The results showed that the expression of these genes changed significantly under stress treatments. Under salt stress, 21 differentially expressed *AhVQ* genes were identified from the salt stress transcriptome data of peanut roots, with 20 of the 21 genes significantly upregulated in roots (**Figure 7**). Notably, *AhVQ11*, *AhVQ15*, and *AhVQ34* showed a marked upregulation, with their expression levels exceeding those of the control group by more than 5-fold. These results suggest that *AhVQ11*, *AhVQ15*, and *AhVQ34* respond to salt stress and may contribute to salt stress tolerance. Under drought stress, eight *AhVQ* genes were identified from the drought stress transcriptome data of peanut roots. All eight responsive *AhVQ* genes were significantly upregulated, with *AhVQ4* exhibiting the most significant induction (17-fold higher expression than the control). These findings indicate that *AhVQ* genes participate in the response of peanut to salt and drought stresses and may play important roles in stress tolerance. It should be noted that PEG treatment induces rapid osmotic shock rather than authentic soil drought, representing a methodological limitation in this study. Under cold treatment, five *AhVQ* genes were identified from the low-temperature stress transcriptome data of peanut leaves, and their expression levels were detected in leaves. Two genes (*AhVQ31* and *AhVQ67*) were upregulated, while three genes (*AhVQ33*, *AhVQ45*, and *AhVQ69*) were downregulated. For all *AhVQ* genes, the expression in response to cold stress was less pronounced than that in response to drought and salt stresses. Only *AhVQ67* showed the most significant upregulated expression (3.2-fold). Notably, members within the same cluster exhibited consistent expression patterns. For instance, *AhVQ15* and *AhVQ34* clustered in subgroup III, while *AhVQ31* and *AhVQ67* were assigned to subgroup VI, suggesting that proteins within the same group may exhibit similar functions.

**Figure 8:**
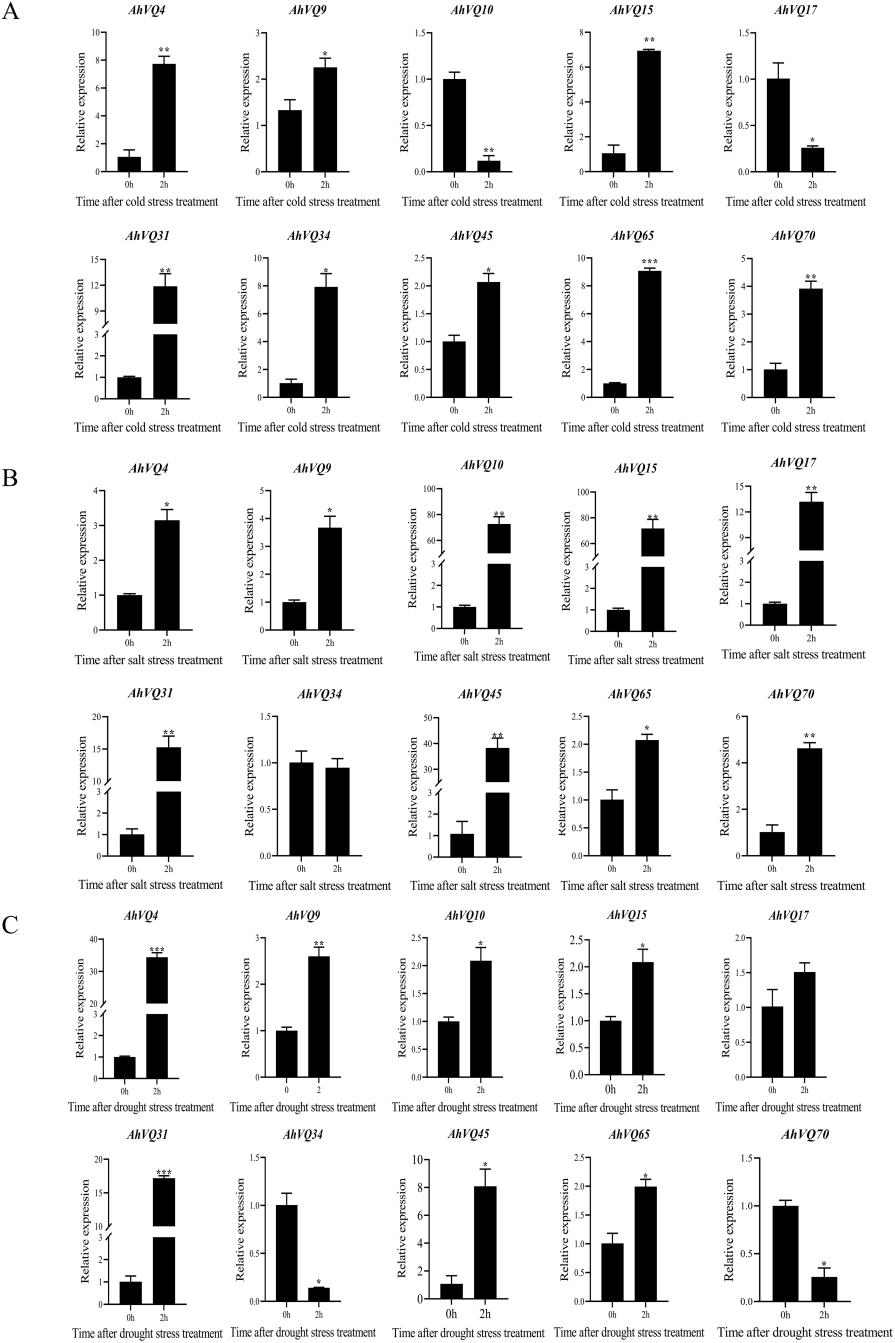
qRT-PCR analysis of *AhVQ* gene expression profiles under diverse abiotic stress conditions. **(A)** Analysis of expression patterns under cold stress. **(B)** Analysis of expression patterns under salt stress. **(C)** Analysis of expression patterns under drought stress. The 0 h column represents the control group, and the 2 h column represents the treatment group. Asterisks indicate statistically significant differences between the treatment and control groups, determined by Student’s t-test. Significance levels are indicated as follows: *P < 0.05, **P < 0.01, ***P < 0.001.

To conduct a more comprehensive analysis of potential functions and to validate transcriptomic gene expression data for the *AhVQ* gene family in various tissues of peanut, we specifically selected and evaluated 10 representative *AhVQ* genes demonstrating the most significant differential expression in transcriptomic analyses (**Figure 8**). Quantitative real-time PCR analysis was performed for these genes in roots and leaves. Under cold stress, all *AhVQ* genes showed significant responses. Among them, seven genes (*AhVQ9*, *AhVQ15*, *AhVQ31*, *AhVQ34*, *AhVQ45*, *AhVQ65*, and *AhVQ70*) were significantly upregulated in leaf tissues at 2 h. Compared to the control group, the expression of these genes increased by approximately 2.1-, 6.6-, 11.9-, 7.9-, 2.1-, 9.1-, and 3.8-fold, respectively. Three genes (*AhVQ4*, *AhVQ10*, and *AhVQ17*) were downregulated under cold stress, suggesting their involvement in negative regulation. Under drought stress, *AhVQ* genes showed varied expression patterns. *AhVQ4*, *AhVQ9*, *AhVQ10*, *AhVQ15*, *AhVQ17*, *AhVQ31*, *AhVQ45*, and *AhVQ65* were significantly upregulated (1.5- to 34-fold increase), while *AhVQ34* and *AhVQ70* were downregulated, indicating rapid regulation in response to drought stress. The expression levels of almost all *AhVQ* genes increased significantly under salt stress, with *AhVQ10* reaching the maximum increase of more than 73-fold.

Transcriptome and qRT-PCR analyses revealed that AhVQ4, AhVQ9 and AhVQ65 participated in drought response. AhVQ9, AhVQ15, AhVQ34 and AhVQ70 were involved in salt tolerance, while AhVQ31 functioned in cold response. These genes serve as elite genetic resources for improving stress tolerance in peanut. Clear discrepancies were observed between qRT-PCR and transcriptome data. *AhVQ17* and *AhVQ34* exhibited opposite expression trends under salt stress, and *AhVQ45* showed inconsistent expression under cold stress. Such divergent patterns were stably detected in three independent replicates. These mismatches were primarily attributed to alternative splicing or reduced primer specificity caused by homologous gene copies. On the whole, the expression profiles of most *AhVQ* genes were consistent with transcriptome data.

### Interaction network of AhVQ proteins

**Figure 9:**
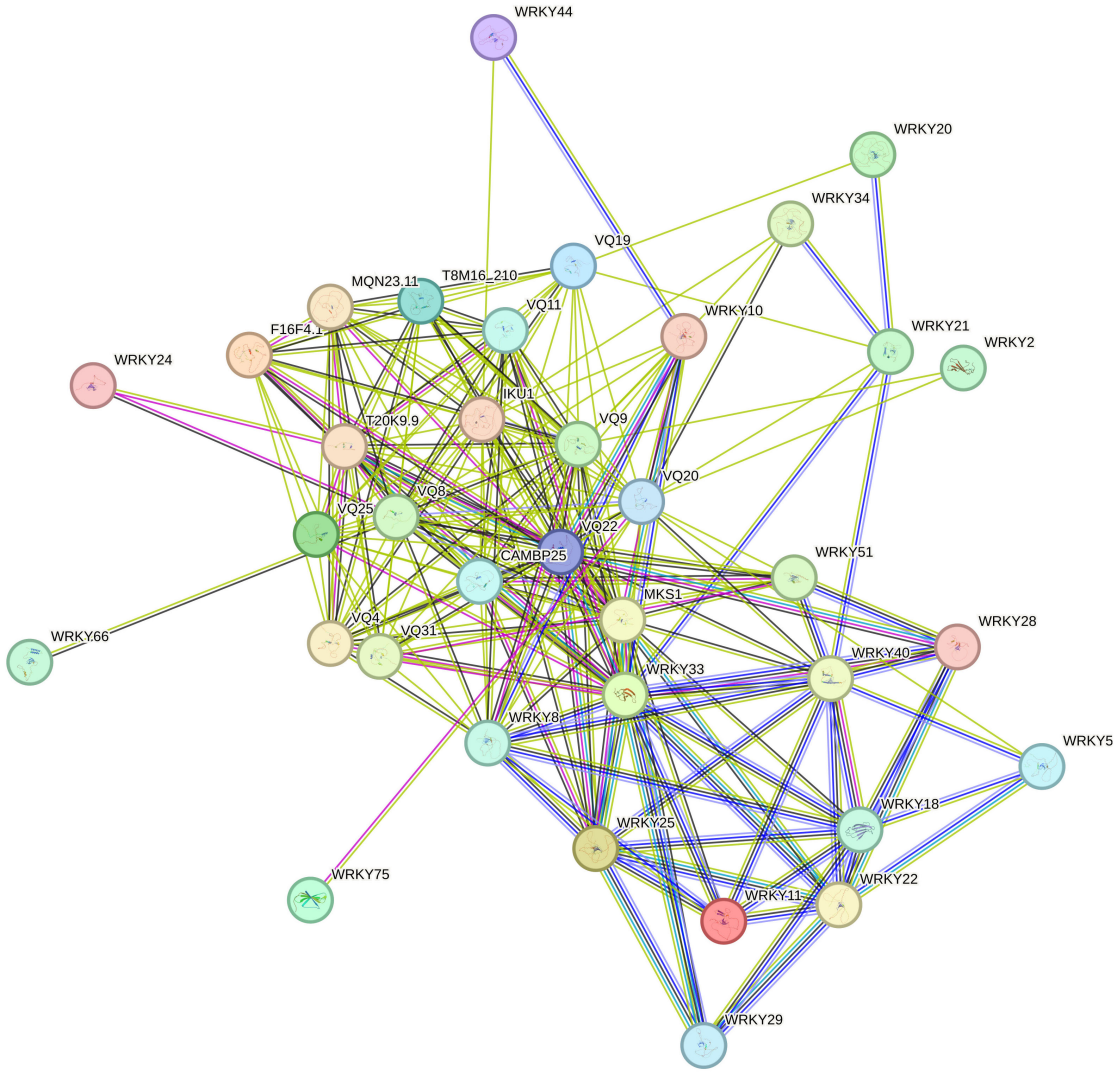
Interaction of AtVQ and AtWRKY proteins in *Arabidopsis thaliana*. Functional and physical interactions of 30 *Arabidopsis* VQ proteins related to AhVQ proteins were validated by constructing an *Arabidopsis* association model.

**Figure 10:**
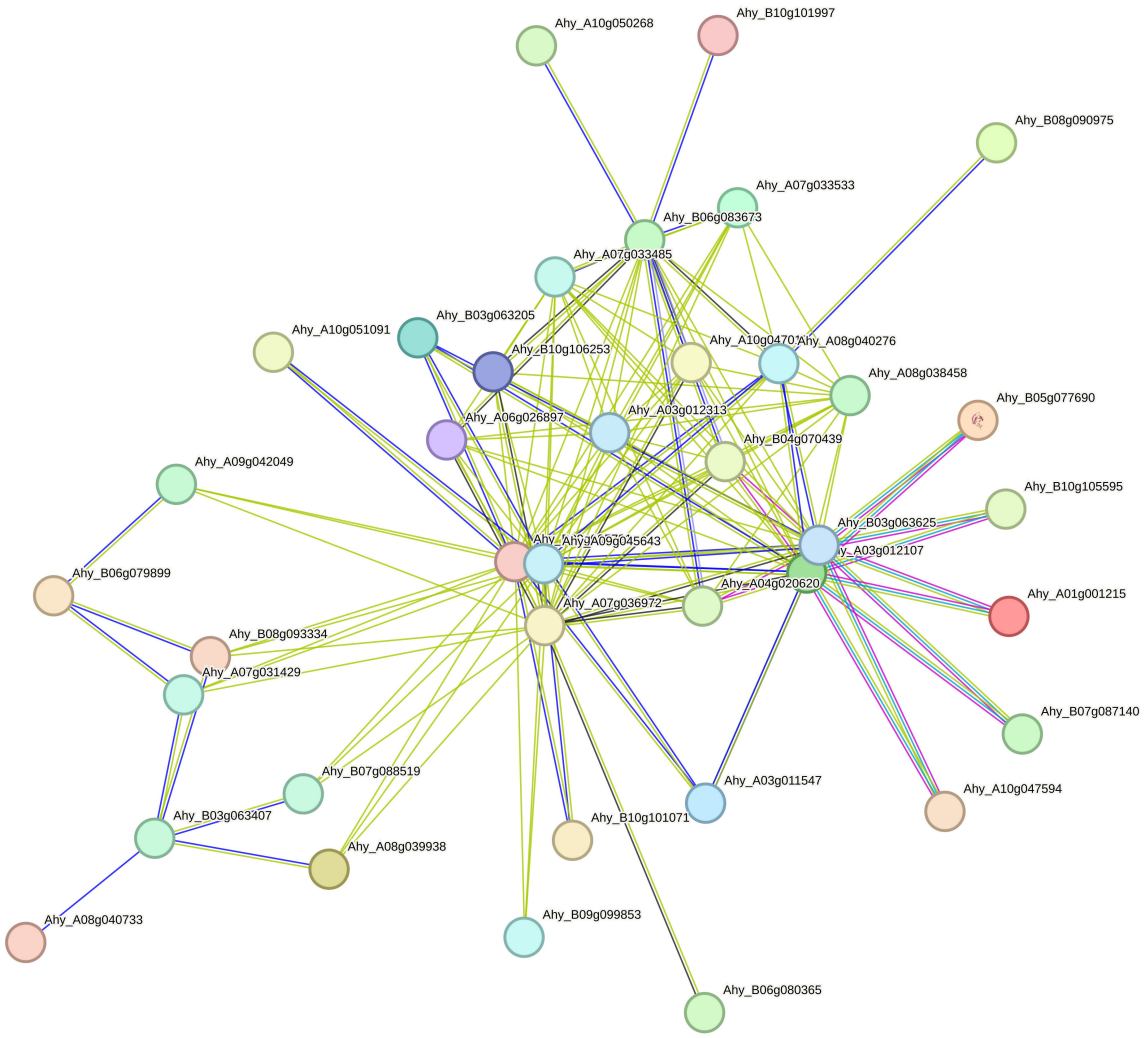
Interaction of AhVQ and AhWRKY proteins in peanut. Functional interacting network models were integrated using the STRING tool. Purple and light blue lines represent known interactions determined from experiments and databases, respectively. Red, green, and blue lines represent predicted interactions from gene fusion, proximity, and co-occurrence, respectively. Black, light green, and gray lines represent other interactions from co-expression, text mining, and protein homology, respectively.

In silico protein–protein interaction analysis was performed using the STRING database based on homology to *Arabidopsis thaliana*, without experimental validation in peanut. To infer potential interaction relationships between AhVQ and AhWRKY proteins, we constructed an *Arabidopsis*-based association model, which provides only predicted functional linkages (**Figure 9**). Two core nodes, AtVQ4 and AtVQ9, exhibited intricate interactions with multiple AtWRKY TFs, suggesting that these AtVQ proteins play critical roles in mediating cellular processes, signaling cascades, and stress responses (**Supplementary Table 4**) (Zhu et al., 2020; Meng et al., 2023). Given that homologous genes tend to share similar functions, the functions of *AhVQ* genes were preliminarily predicted based on the interaction patterns between AtVQ and AtWRKY proteins in *Arabidopsis*. Subsequently, we constructed a peanut-specific protein interaction model to predict the interaction relationships among AhVQ proteins and between AhVQ and AhWRKY proteins. The results showed that 30 AhVQ proteins, homologous to functionally characterized AtVQ proteins, were mapped into predicted interaction networks (**Supplementary Table 5**; **Figure 10**). These *in silico* results suggest potential regulatory roles for these AhVQ proteins in growth, development, and stress responses, pending further experimental validation. Notably, according to the *in silico* predictions, AhVQ proteins may not function independently, and potential interaction relationships were inferred among multiple *AhVQ* family members. For example, *AhVQ28*, *AhVQ61*, and *AhVQ64*, which are all classified into subgroup IV, were found to interact with each other in the peanut predictive model. This finding suggests that AhVQ proteins within the same phylogenetic group may share similar biological functions and potentially act in a coordinated manner, according to the *in silico* interaction predictions.

## Discussion

The *VQ* family was defined by the conserved FxxhVQxhTG motif and exhibits high evolutionary conservation across taxa. Similar to the *VQ* families in *Arabidopsis*, rice and soybean, AhVQ proteins in peanut retain conserved motif architectures and simplified gene structures, reflecting the universal evolutionary characteristics of this gene family (Cheng et al., 2012; Zheng et al., 2022; Kim et al., 2013). A core amino acid variation (VQ to VH) commonly found in monocots was absent in dicotyledonous peanut, which further reveals the evolutionary divergence of *VQ* genes between monocots and dicots (Kim et al., 2013; Zhang et al., 2022a; Xu et al., 2025). Several key *AhVQ* members, including AhVQ4, *AhVQ15*, *AhVQ31* and *AhVQ65*, possessed intact VQ domains. Their structural conservation is closely linked to stable biological functions in plant stress signaling, as observed for their stress-related homologs in *Arabidopsis*. Most *AhVQ* genes lacked introns. This structural characteristic facilitated rapid transcriptional activation under abiotic stress, which was a common feature of plant stress-responsive genes.

The prevalence of intronless genes in *AhVQ* genes may facilitate rapid transcriptional induction in response to abiotic stress, a structural feature widely recognized for its association with stress responsiveness in plants. The high proportion of intronless genes (83.1%) in *AhVQ* genes is a typical feature of the plant *VQ* family (Kim et al., 2013; Dong et al., 2018; Ding et al., 2019; Wang et al., 2019a; Zheng et al., 2022; Xu et al., 2025). Intron loss was a major evolutionary adaptation for plants to achieve rapid stress responses (Roy and Penny, 2007). Phylogenetic analyses demonstrate that VQ proteins from dicots and monocots form distinct clades, which aligns with the general evolutionary trajectory of land plants (Jiang et al., 2018). As a dicot species, AhVQs showed closer phylogenetic relationships with *Arabidopsis* VQs than with rice VQs. The tight clustering of stress-related orthologous pairs between peanut and *Arabidopsis* further proves that the stress regulatory roles of *VQ* genes originated prior to species differentiation and have been well conserved during evolution. The conserved core motifs and diversified accessory sequences among AhVQ subgroups also support the coexistence of functional conservation and functional differentiation within this gene family (Fan et al., 2026). In line with this evolutionary conservation, *AhVQ4*, *AhVQ15*, *AhVQ31*, and *AhVQ65* are distributed in different subgroups but maintain similar stress-related structural characteristics. In this study, we determined the expression levels of three pairs of phylogenetically closely clustered *AhVQ* genes (*AhVQ31*/*AhVQ67*, *AhVQ4*/*AhVQ9*, and *AhVQ15*/*AhVQ34*) under low temperature (4°C), drought (20% PEG-6000), and salt (1% NaCl) treatments. *AhVQ4* and *AhVQ9* were clustered into subgroup II with *AtVQ12* (*AT2G22880*). *AhVQ31* and *AhVQ67* were clustered into subgroup VI with *MVQ1* (*AT1G28280*), which acts as a negative regulator of mitogen-activated protein kinase (MAPK) cascades (Pecher et al., 2014). *AhVQ15* and *AhVQ34* in subgroup III were closely related to *AtVQ20*; these two *AhVQ* genes displayed similar expression levels and were highly expressed in roots, suggesting that they may have similar functions.

Multiple stress and hormone-related cis-elements were detected in the promoter regions of *AhVQ* genes, which largely determine their stress-responsive expression profiles (Hu et al., 2026; Sun et al., 2026). The promoters of *AhVQ31* and *AhVQ67* (homologs of *AtVQ4*) harbored ABRE and LTR cis-acting elements. ABRE was a core component of the ABA signaling pathway. It responded to ABA signals and modulates gene expression under abiotic stress (Liu J. et al., 2026; Yu et al., 2026; Liu W. et al., 2026). LTR participates in cold stress response. It regulates cold-tolerant gene expression and improves plant cold resistance. In this study, the two genes were highly expressed and significantly upregulated under cold stress. The results indicated that *AhVQ31* and *AhVQ67* were ideal candidate genes for cold stress tolerance (Hussain et al., 2026). This regulatory mode was highly conserved among different plant species, as similar correlations between cis-element distribution and gene stress responses had been reported in wheat, mustard and tobacco (Zhang et al., 2022b; Zheng et al., 2022; Liu et al., 2020). Combined with existing research on plant *VQ* genes, we infer that *AhVQ33* and *AhVQ67* act as important regulators in peanut cold tolerance. The expressions of *AhVQ4*, *AhVQ44*, *AhVQ22*, *AhVQ48*, *AhVQ9*, *AhVQ55*, *AhVQ13*, and *AhVQ65* increased under drought treatment, with *AhVQ4* showing the most significant increase. *AhVQ4* and *OsVQ24* belong to the same group. Previous studies on rice *VQs* showed that the expression of *OsVQ24* increased within 2 h under drought treatment (Xixu et al., 2020). Interestingly, in this study, the expression of all differentially expressed *AhVQ* genes increased under drought stress. *AhVQ4* showed the strongest induction, implying that it acts as a core drought regulator inherited from ancestral stress modules. Previous studies, such as those on *TaVQ4-D*, have suggested that it up-regulates the expression of ROS-scavenging genes and stress-related genes in wheat under drought stress (Tian et al., 2023b). Another example is the effect of *MDVQ37* on the SA pathway, resulting in changes in drought tolerance, which suggests that *VQ* genes play an important role in drought tolerance in many species (Dong et al., 2022). Furthermore, other reports found that the majority of the *VQ* genes were upregulated under drought stress in cotton and sweet potato (Chen et al., 2020; Si et al., 2023). Therefore, we speculated that *AhVQ* genes play an important role in drought stress in peanut. *AtVQ9* is a well-known root-specific regulator of salt tolerance in *Arabidopsis*. Our orthology data confirmed that *AhVQ23* and *AhVQ56* are the confirmed peanut orthologs of *AtVQ9*. These two *AhVQ* genes showed root-preferential expression and strong salt induction, fully mirroring the pattern of *AtVQ9*. This cross-species consistency strongly suggests that the salt-tolerance module represented by *AtVQ9* is functionally conserved in peanut. Unlike *AtVQ9*, *AhVQ23*/*AhVQ56* also responded to drought, indicating neofunctionalization during peanut adaptation to multiple stresses. Exposure to drought and salt stress induced the accumulation of hydrogen peroxide and markedly decreased antioxidant enzymes, thereby impairing plant growth and development (Magwanga et al., 2019; Xia et al., 2021). These results indicated that the cis-element prediction was accurate and could effectively reflect the real stress response patterns of *AhVQ* genes. We found that *AhVQ9*, *AhVQ13*, *AhVQ22*, *AhVQ44*, *AhVQ48*, *AhVQ55*, and *AhVQ65* showed an upward trend under drought and salt treatments, indicating that *AhVQ* genes are actively involved in the response of peanut to salt and drought stress. The RNA-seq and qRT-PCR results showed broad consistency at the plant tissue level. In conclusion, *VQ*-related genes are involved in a variety of stress-related responses and play important roles.

VQ proteins interact with multiple TFs to modulate diverse physiological processes, facilitating proper responses to abiotic stress (Tian et al., 2023b). Among these, WRKY proteins are generally considered the most important interaction partners of VQ proteins (Li et al., 2021). For example, *SlVQ16* positively regulated tomato tolerance to salt (Ma et al., 2023), and *MaVQ5* physically interacted with *MaWRKY26*, thereby attenuating *MaWRKY26* response to cold stress. To elucidate the interaction relationships between VQ proteins and other proteins, an *Arabidopsis* association model was constructed. This model revealed a complex VQ-WRKY interaction network with two central hubs, AtVQ4 and AtVQ9. Transcriptomic and qRT-PCR analyses demonstrated that the expression levels of AtVQ4 and AtVQ9 were upregulated under multiple abiotic stresses, suggesting their important roles in stress responses. Correspondingly, AhVQ4 and AhVQ31 also showed strong interactions with multiple proteins in the peanut network, which may explain their significant responses under multiple stresses. The VQ-WRKY module shows high conservation between *Arabidopsis* and peanut (Uji et al., 2019; Yu et al., 2019). AtVQ9 binds WRKY8 to modulate salt tolerance. Its homologous proteins, AhVQ23 and AhVQ56, in peanut can also interact with AhWRKY members. This finding indicates the existence of a conserved inhibitory regulatory module in peanut roots. AtVQ4 interacts with WRKY33 to participate in stress response processes. Its peanut orthologs, AhVQ31 and AhVQ67, serve as key interaction nodes in the regulatory network. These results further validate the conservation of this core stress signaling module. Nevertheless, the present study was limited to bioinformatics prediction and transcriptome expression analysis. The physical interaction between AhVQ and AhWRKY proteins, as well as the downstream regulatory pathways, remained to be verified via *in vitro* and *in vivo* experiments. In addition, the functional differentiation of homeologous *AhVQ* gene pairs in this allotetraploid peanut had not been fully clarified. Further genetic transformation and phenotypic identification were required to uncover the molecular mechanisms of key *AhVQ* genes in stress tolerance.

## Conclusions

A total of 71 *AhVQ* genes were identified in cultivated peanut in this work. Analyses of their sequence features, including conserved motifs, functional domains and promoter regions, revealed the molecular characteristics of the *AhVQ* family. Phylogenetic tree and collinearity analyses demonstrated that segmental and tandem duplications were the major driving forces underlying the evolution and expansion of *AhVQ* genes in cultivated peanut. Transcriptome-based expression profiling showed genes, including *AhVQ31*, were constitutively expressed across tissues, whereas genes such as *AhVQ65* exhibited tissue-preferential expression patterns. Multiple *AhVQ* genes represented by *AhVQ4* were strongly induced under drought stress; several members including *AhVQ65* participated in salt stress responses, and a set of genes such as *AhVQ31* contributed to cold stress adaptation. In silico interaction prediction further uncovered extensive crosstalk between AhVQ proteins and WRKY transcription factors, laying a solid groundwork for future functional characterization of these genes. Collectively, this study not only establishes a fundamental basis for exploring the biological functions of *AhVQs*, but also provides valuable insights into the evolutionary dynamics of peanut genomes.

## Data Availability

The datasets presented in this study can be found in online repositories. The names of the repository/repositories and accession number(s) can be found below: https://www.ncbi.nlm.nih.gov/, PRJNA1335572.

## Supplementary material

Supplementary Figure 1 Multiple sequence alignment of the conserved VQ motif in AhVQ proteins.

Supplementary Table 1 qRT-PCR primer sequences.

Supplementary Table 2 *VQ* gene family members in cultivated peanut.

Supplementary Table 3 Sequence logos for the conserved motifs within VQ proteins.

Supplementary Table 4 Information regarding the STRING search results of the interaction networks of AtVQ proteins.

Supplementary Table 5 Information regarding the STRING search results of the interaction networks of AhVQ proteins.

## References

[B1] Bailey T. L. Boden M. Buske F. A. Frith M. Grant C. E. Clementi L. . (2009). MEME SUITE: tools for motif discovery and searching. Nucleic Acids 37, W202–W208. doi: 10.1093/nar/gkp335 19458158 PMC2703892

[B2] Chen C. Chen H. Zhang Y. Thomas H. R. Frank M. H. He Y. . (2020). TBtools: An integrative toolkit developed for interactive analyses of big biological data. Mol. Plant 13, 1194–1202. doi: 10.1016/j.molp.2020.06.009 32585190

[B3] Chen S. Cao H. Huang B. Zheng X. Liang K. Wang G. L. . (2022). The WRKY10-VQ8 module safely and effectively regulates rice thermotolerance. Plant Cell Environ. 45, 2126–2144. doi: 10.1111/pce.14329 35394666

[B4] Cheng Y. Zhou Y. Yang Y. Chi Y. J. Zhou J. Chen J. Y. . (2012). Structural and functional analysis of VQ motif-containing proteins in Arabidopsis as interacting proteins of WRKY transcription factors. Plant Physiol. 159, 810–825. doi: 10.1104/pp.112.196816 22535423 PMC3375943

[B5] Clevenger J. Chu Y. Scheffler B. Ozias-Akins P. (2016). A developmental transcriptome map for allotetraploid Arachis hypogaea. Front. Plant Sci. 7, 1446. doi: 10.3389/fpls.2016.01446 27746793 PMC5043296

[B6] de Camargo Santos A. Schaffer B. Ioannou A. G. Moon P. Shahid M. Rowland D. . (2024). Melatonin seed priming improves early establishment and water stress tolerance of peanut. Plant Physiol. Biochem. 211, 108664. doi: 10.1016/j.plaphy.2024.108664 38703498

[B7] Ding H. Yuan G. Mo S. Qian Y. Wu Y. Chen Q. . (2019). Genome-wide analysis of the plant-specific VQ motif-containing proteins in tomato (Solanum lycopersicum) and characterization of SlVQ6 in thermotolerance. Plant Physiol. Biochem. 143, 29–39. doi: 10.1016/j.plaphy.2019.08.019 31479880

[B8] Dong J. Chen C. Chen Z. (2003). Expression profiles of the Arabidopsis WRKY gene superfamily during plant defense response. Plant Mol. Biol. 51, 21–37. doi: 10.1023/a:1020780022549 12602888

[B9] Dong Q. Duan D. Zheng W. Huang D. Wang Q. Yang J. . (2022). Overexpression of MdVQ37 reduces drought tolerance by altering leaf anatomy and SA homeostasis in transgenic apple. Tree Physiol. 42, 160–174. doi: 10.1093/treephys/tpab098 34328189

[B10] Dong Q. Zhao S. Duan D. Tian Y. Wang Y. Mao K. . (2018). Structural and functional analyses of genes encoding VQ proteins in apple. Plant Sci. 272, 208–219. doi: 10.1016/j.plantsci.2018.04.029 29807593

[B11] Fan Z. Liu Z. Wan X. Zheng T. Pan J. Huang R. . (2026). Genome-wide identification and expression profiling of the CoVQ gene family in Camellia Oleifera. BMC Plant Biol. 26, 436. doi: 10.1186/s12870-026-08227-0 41639777 PMC12964900

[B12] Gasteiger E. Gattiker A. Hoogland C. Ivanyi I. Appel R. D. Bairoch A. (2003). ExPASy: The proteomics server for in-depth protein knowledge and analysis. Nucleic Acids Res. 31, 3784–3788. doi: 10.1093/nar/gkg563 12824418 PMC168970

[B13] Gundaraniya S. A. Ambalam P. S. Budhwar R. Padhiyar S. M. Tomar R. S. (2023). Transcriptome analysis provides insights into the stress response in cultivated peanut (Arachis hypogaea L.) subjected to drought-stress. Mol. Biol. Rep. 50, 6691–6701. doi: 10.1007/s11033-023-08563-6 37378750

[B14] Hao Z. Tian J. Fang H. Fang L. Xu X. He F. . (2022). A VQ-motif-containing protein fine-tunes rice immunity and growth by a hierarchical regulatory mechanism. Cell Rep. 40, 111235. doi: 10.1016/j.celrep.2022.111235 35977497

[B15] He Q. He M. Zhang X. Zhang X. Zhang W. Dong J. . (2023). RsVQ4-RsWRKY26 module positively regulates thermotolerance by activating RsHSP70-20 transcription in radish (Raphanus sativus L.). Environ. Exp. Bot. 214, 105467. doi: 10.1016/j.envexpbot.2023.105467 38826717

[B16] Hu Y. Chen L. Wang H. Zhang L. Wang F. Yu D. (2013). Arabidopsis transcription factor WRKY8 functions antagonistically with its interacting partner VQ9 to modulate salinity stress tolerance. Plant J. 74, 730–745. doi: 10.1111/tpj.12159 23451802

[B17] Hu B. Jin J. Guo A. Y. Zhang H. Luo J. Gao G. (2015). GSDS 2.0: an upgraded gene feature visualization server. Bioinformatics 31, 1296–1297. doi: 10.1093/bioinformatics/btu817 25504850 PMC4393523

[B18] Hu Z. Qian R. Xie F. Wang Z. Yan P. Yang J. . (2026). Comparative genomics and expression analysis of the CIPK gene family in rice (Oryza sativa) and foxtail millet (Setaria italica). Front Plant Sci. 16, 1710663. doi: 10.3389/fpls.2025.1710663 41560925 PMC12812626

[B19] Hussain S. Jing X. Tariq F. Wang X. Wang W. Li G. . (2026). Genome-wide identification and functional characterization of ABA and salt stress responsive AITRs transcriptional repressors genes in sweet potato (Ipomoea batatas). BMC Plant Biol. doi: 10.1186/s12870-026-08963-3 42151792 PMC13352788

[B20] Jiang S. Y. Sevugan M. Ramachandran S. (2018). Valine-glutamine (VQ) motif-containing genes are ancient and non-plant-specific with comprehensive expression regulation by various biotic and abiotic stresses. BMC Genomics 19, 342. doi: 10.1186/s12864-018-4733-7 29743038 PMC5941492

[B21] Jing Y. Lin R. (2015). The VQ motif-containing protein family of plant-specific transcriptional regulators. Plant Physiol. 169, 371–378. doi: 10.1104/pp.15.00788 26220951 PMC4577417

[B22] Kim D. Y. Kwon S. I. Choi C. Lee H. Ahn I. Park S. R. . (2013). Expression analysis of rice VQ genes in response to biotic and abiotic stresses. Gene 529, 208–214. doi: 10.1016/j.gene.2013.08.023 23958655

[B23] Kumar S. Stecher G. Tamura K. (2016). MEGA7: molecular evolutionary genetics analysis version 7.0 for bigger datasets. Mol. Biol. Evol. 33, 1870–1874. doi: 10.1093/molbev/msw054 27004904 PMC8210823

[B24] Lescot M. Déhais P. Thijs G. Marchal K. Moreau Y. Van de Peer Y. . (2002). PlantCARE, a database of plant cis-acting regulatory elements and a portal to tools for in silico analysis of promoter sequences. Nucleic Acids Res. 30, 325–327. doi: 10.1093/nar/30.1.325 11752327 PMC99092

[B25] Li N. Li X. Xiao J. Wang S. (2014). Comprehensive analysis of VQ motif-containing gene expression in rice defense responses to three pathogens. Plant Cell Rep. 33, 1493–1505. doi: 10.1007/s00299-014-1633-4 24871256

[B26] Li C. Yan C. Sun Q. Wang J. Yuan C. Mou Y. . (2021). The bHLH transcription factor AhbHLH112 improves the drought tolerance of peanut. BMC Plant Biol. 21, 540. doi: 10.1186/s12870-021-03318-6 34784902 PMC8594184

[B27] Liu C. Liu H. Zhou C. Timko M. P. (2020). Genome-wide identification of the VQ protein gene family of tobacco (Nicotiana tabacum L.) and analysis of its expression in response to phytohormones and abiotic and biotic stresses. Genes (Basel) 11 (3), 284. doi: 10.3390/genes11030284 32156048 PMC7140788

[B28] Liu J. Yu Z. Hou X. Wang J. Fu X. Zhang H. . (2026). Functional characterization of TaSnRK2.8-5A reveals the central role of its signal module in enhancing drought tolerance through coordinated molecular and physiological response of wheat. Plant Physiol Biochem. 235, 111397. doi: 10.1016/j.plaphy.2026.111397 42160837

[B29] Liu W. Liu H. Liu X. Yue Z. Yan J. Wei L. . (2026). Molecular mechanism of cold acclimation regulating freezing tolerance in prunus mume. Plant Physiol. 3, kiag333. doi: 10.1093/plphys/kiag333 PMC13316940 42234825

[B30] Liu H. Xing M. Yang W. Mu X. Wang X. Lu F. . (2019). Genome-wide identification of and functional insights into the late embryogenesis abundant (LEA) gene family in bread wheat (Triticum aestivum). Sci. Rep. 9, 13375. doi: 10.1038/s41598-019-49759-w 31527624 PMC6746774

[B31] Livak K. J. Schmittgen T. D. (2001). Analysis of relative gene expression data using real-time quantitative PCR and the 2(-Delta Delta C(T)) Method. Methods 25, 402–408. doi: 10.1006/meth.2001.1262 11846609

[B32] Ma J. Li C. Sun L. Ma X. Qiao H. Zhao W. . (2023). The SlWRKY57-SlVQ21/SlVQ16 module regulates salt stress in tomato. J. Integr. Plant Biol. 65, 2437–2455. doi: 10.1111/jipb.13562 37665103

[B33] Magwanga R. O. Lu P. Kirungu J. N. Dong Q. Cai X. Zhou Z. . (2019). Knockdown of cytochrome P450 genes Gh_D07G1197 and Gh_A13G2057 on chromosomes D07 and A13 reveals their putative role in enhancing drought and salt stress tolerance in Gossypium hirsutum. Genes (Basel) 10 (3), 226. doi: 10.3390/genes10030226 30889904 PMC6471685

[B34] Meng X. Lu M. Xia Z. Li H. Liu D. Li K. . (2023). Wheat VQ motif-containing protein VQ25-A facilitates leaf senescence via the abscisic acid pathway. Int. J. Mol. Sci. 24 (18), 13839. doi: 10.3390/ijms241813839 37762142 PMC10531066

[B35] Pecher P. Eschen-Lippold L. Herklotz S. Kuhle K. Naumann K. Bethke G. . (2014). The Arabidopsis thaliana mitogen-activated protein kinases MPK3 and MPK6 target a subclass of 'VQ-motif'-containing proteins to regulate immune responses. New Phytol. 203, 592–606. doi: 10.1111/nph.12817 24750137

[B36] Roy S. W. Penny D. (2007). Patterns of intron loss and gain in plants: intron loss-dominated evolution and genome-wide comparison of O. sativa and A. thaliana. Mol. Biol. Evol. 24, 171–181. doi: 10.1093/molbev/msl159 17065597

[B37] Si Z. Wang L. Ji Z. Qiao Y. Zhang K. Han J. (2023). Genome-wide comparative analysis of the valine glutamine motif containing genes in four Ipomoea species. BMC Plant Biol. 23, 209. doi: 10.1186/s12870-023-04235-6 37085761 PMC10122360

[B38] Song H. Wang P. Lin J. Y. Zhao C. Bi Y. Wang X. (2016). Genome-wide identification and characterization of WRKY gene family in peanut. Front. Plant Sci. 7, 534. doi: 10.3389/fpls.2016.00534 27200012 PMC4845656

[B39] Sun Y. Wang J. Yu Y. Wu L. Ruan B . (2026). Molecular and physiological regulation of premature leaf senescence in rice. Plants (Basel). 15 (6), 869. doi: 10.3390/plants15060869 41901388 PMC13030633

[B40] Song W. Zhao H. Zhang X. Lei L. Lai J. (2015). Genome-wide identification of VQ motif-containing proteins and their expression profiles under abiotic stresses in maize. Front. Plant Sci. 6, 1177. doi: 10.3389/fpls.2015.01177 26779214 PMC4700186

[B41] Szklarczyk D. Gable A. L. Nastou K. C. Lyon D. Kirsch R. Pyysalo S. . (2021). The STRING database in 2021: customizable protein-protein networks, and functional characterization of user-uploaded gene/measurement sets. Nucleic Acids Res. 49, d605–d612. doi: 10.1093/nar/gkaa1074 33237311 PMC7779004

[B42] Tian J. Zhang J. Francis F. (2023a). Large-scale identification and characterization analysis of VQ family genes in plants, especially gymnosperms. Int. J. Mol. Sci. 24 (19), 14968. doi: 10.3390/ijms241914968 37834416 PMC10573558

[B43] Tian J. Zhang J. Francis F. (2023b). The role and pathway of VQ family in plant growth, immunity, and stress response. Planta 259, 16. doi: 10.1007/s00425-023-04292-z 38078967

[B44] Uji Y. Kashihara K. Kiyama H. Mochizuki S. Akimitsu K. Gomi K. (2019). Jasmonic acid-induced VQ-motif-containing protein OsVQ13 influences the OsWRKY45 signaling pathway and grain size by associating with OsMPK6 in rice. Int. J. Mol. Sci. 20 (12), 2917. doi: 10.3390/ijms20122917 31207967 PMC6627515

[B45] Wang Y. Jiang Z. Li Z. Zhao Y. Tan W. Liu Z. . (2019a). Genome-wide identification and expression analysis of the VQ gene family in soybean (Glycine max). PeerJ 7, e7509. doi: 10.7717/peerj.7509 31497394 PMC6708371

[B46] Wang Y. Li J. Li M. Li Y. Zhao Z. Li C. . (2022). Genome-wide characterization of remorin genes in terms of their evolution and expression in response to hormone signals and abiotic stresses in foxtail millet (Setaria italica). Diversity 14, 711. doi: 10.3390/d14090711 30654563

[B47] Wang M. Vannozzi A. Wang G. Zhong Y. Corso M. Cavallini E. . (2015). A comprehensive survey of the grapevine VQ gene family and its transcriptional correlation with WRKY proteins. Front. Plant Sci. 6, 417. doi: 10.3389/fpls.2015.00417 26124765 PMC4464145

[B48] Wang Z. Yan L. Wan L. Huai D. Kang Y. Shi L. . (2019b). Genome-wide systematic characterization of bZIP transcription factors and their expression profiles during seed development and in response to salt stress in peanut. BMC Genomics 20, 51. doi: 10.1186/s12864-019-5434-6 30651065 PMC6335788

[B49] Wang Y. Zhang M. Wu C. Chen C. Meng L. Zhang G. . (2024). SlWRKY51 regulates proline content to enhance chilling tolerance in tomato. Plant Cell Environ. 47, 5104–5114. doi: 10.1111/pce.15081 39148214

[B50] Xia Y. Yang J. Ma L. Yan S. Pang Y. (2021). Genome-wide identification and analyses of drought/salt-responsive cytochrome P450 genes in Medicago truncatula. Int. J. Mol. Sci. 22 (18), 9957. doi: 10.3390/ijms22189957 34576120 PMC8467197

[B51] Xixu P. Ting X. Jiao M. Zong T. Dinggang Z. Xinke T. . (2020). Differential expression of rice valine-glutamine gene family in response to nitric oxide and regulatory circuit of OsVQ7 and OsWRKY24. Rice Sci. 27, 10–20. doi: 10.1016/j.rsci.2019.12.002 38826717

[B52] Xu Y. Zhang Z. Ding H. Wen S. Zhang G. Qin F. . (2021). Comprehensive effects of salt stress and peanut cultivars on the rhizosphere bacterial community diversity of peanut. Arch. Microbiol. 204, 15. doi: 10.1007/s00203-021-02619-6 34894277

[B53] Xu J. Zhong X. Wang H. Shi H. Zuo G. Yin L. . (2025). Integrative meta-QTL and RNA-Seq analysis reveals valine-glutamine (VQ) motif-containing ZmVQ56 as a key regulator of water-nitrogen interaction in maize (Zea mays L.). Int. J. Biol. Macromol. 311, 143353. doi: 10.1016/j.ijbiomac.2025.143353 40274169

[B54] Yu T. Lu X. Bai Y. Mei X. Guo Z. Liu C. . (2019). Overexpression of the maize transcription factor ZmVQ52 accelerates leaf senescence in Arabidopsis. PloS One 14, e0221949. doi: 10.1371/journal.pone.0221949 31469881 PMC6716648

[B55] Yu J. Zhang X. Wang S. Wang D. Gao Y. Li X. . (2026). Genome-wide Identification of NF-YA transcription factors in strawberry and their responses to salt stress. Plants (Basel). 15 (10), 1475. doi: 10.3390/plants15101475 42197610 PMC13211191

[B56] Yuan C. Sun Q. Kong Y . (2019). Genome-wide mining seed-specific candidate genes from peanut for promoter cloning. PLoS One. 14 (3), e0214025. doi: 10.1371/journal.pone.0214025 30921362 PMC6438489

[B57] Yuan C. Li C. Lu X. Zhao X. Yan C. Wang J. . (2020). Comprehensive genomic characterization of NAC transcription factor family and their response to salt and drought stress in peanut. BMC Plant Biol. 20, 454. doi: 10.1186/s12870-020-02678-9 33008287 PMC7532626

[B58] Zhai M. Ao Z. Qu H. Guo D. (2024). Overexpression of the potato VQ31 enhances salt tolerance in Arabidopsis. Front. Plant Sci. 15, 1347861. doi: 10.3389/fpls.2024.1347861 38645398 PMC11027747

[B59] Zhang L. Wang K. Han Y. Yan L. Zheng Y. Bi Z. . (2022a). Genome-wide analysis of the VQ motif-containing gene family and expression profiles during phytohormones and abiotic stresses in wheat (Triticum aestivum L.). BMC Genomics 23, 292. doi: 10.1186/s12864-022-08519-3 35410124 PMC8996428

[B60] Zhang G. Wei B. (2019). Characterization of VQ motif-containing protein family and their expression patterns under phytohormones and abiotic stresses in melon (Cucumis melo L.). Plant Growth Regul. 89, 273–285. doi: 10.1007/s10725-019-00534-x 30311153

[B61] Zhang S. Yu Y. Song T. Zhang M. Li N. Yu M. . (2022b). Genome-wide identification of foxtail millet's TRX family and a functional analysis of SiNRX1 in response to drought and salt stresses in transgenic Arabidopsis. Front. Plant Sci. 13, 946037. doi: 10.3389/fpls.2022.946037 36226299 PMC9549295

[B62] Zhang D. Zhu X. Du X. Wang X. Wang B. Wei X. (2025). Identification of the Valine-Glutamine gene family in Chenopodium quinoa Willd and analysis of its expression pattern and subcellular localization under drought stress. BMC Genomics 26, 252. doi: 10.1186/s12864-025-11313-6 40087573 PMC11908108

[B63] Zheng J. Li H. Guo Z. Zhuang X. Huang W. Mao C. . (2022). Comprehensive identification and expression profiling of the VQ motif-containing gene family in Brassica juncea. Biol. (Basel) 11 (12), 1814. doi: 10.3390/biology11121814 36552323 PMC9776337

[B64] Zhong Y. Wang P. Zhang X. Cheng Z. M. (2021). Recent duplications dominate VQ and WRKY gene expansions in six prunus species. Int. J. Genomics 2021, 4066394. doi: 10.1155/2021/4066394 34961840 PMC8710041

[B65] Zhou Y. Yang Y. Zhou X. Chi Y. Fan B. Chen Z. (2016). Structural and functional characterization of the VQ protein family and VQ protein variants from soybean. Sci. Rep. 6, 34663. doi: 10.1038/srep34663 27708406 PMC5052590

[B66] Zhu H. Zhou Y. Zhai H. He S. Zhao N. Liu Q. (2020). A novel sweetpotato WRKY transcription factor, IbWRKY2, positively regulates drought and salt tolerance in transgenic Arabidopsis. Biomolecules 10 (4), 506. doi: 10.3390/biom10040506 32230780 PMC7226164

