# Supplementary material for: Genome-wide identification and characterization of VQ genes from cultivated peanut and their response to abiotic stresses
Source: Front Plant Sci. 2026 Jul 3;17:1865834. doi: 10.3389/fpls.2026.1865834 (PMC13376307; doi:10.3389/fpls.2026.1865834)
Supplement: Supplementary Figure 1 — Multiple sequence alignment of the conserved VQ motif in AhVQ proteins. [file Image1.pdf]

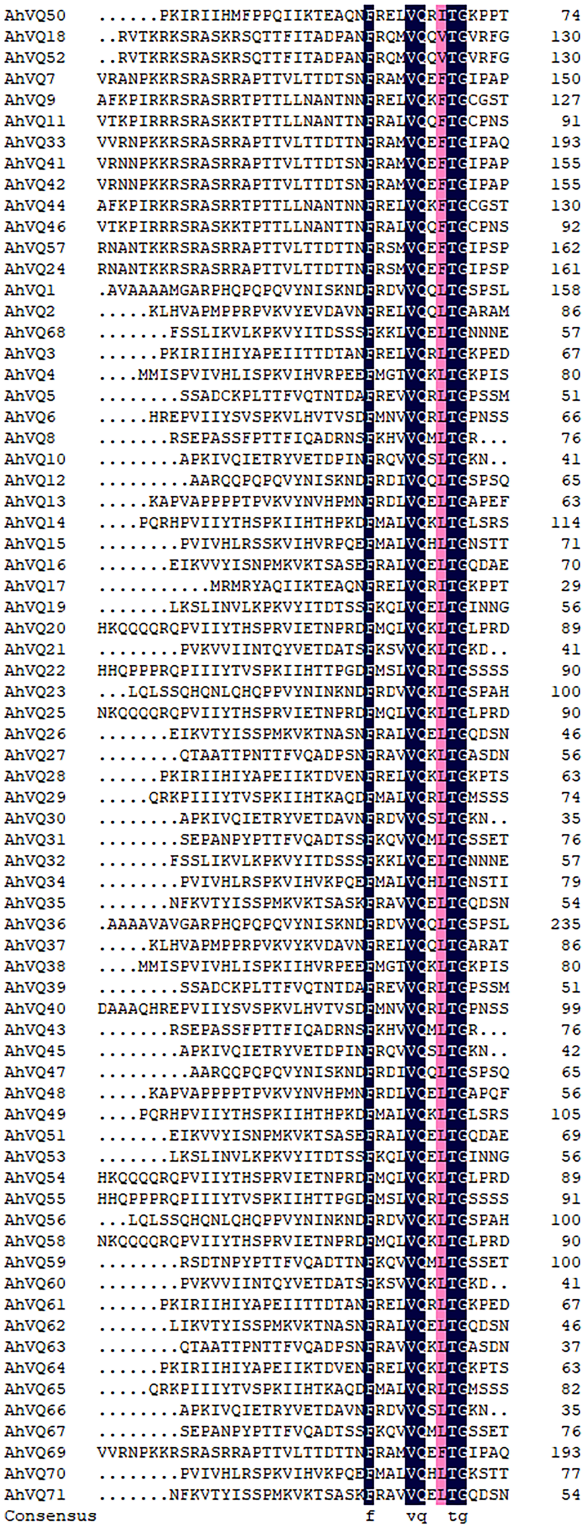

Supplementary Figure S1. Multiple sequence alignment of the conserved VQ domains from *AhVQ* family proteins. Each AhVQ protein identifier is shown on the left, with numerals at the end of each sequence indicating the total amino acid count of the corresponding full-length protein. The bottom Consensus row displays the consensus sequence of the characteristic VQ motif. Dark blue vertical bars highlight the core conserved stretch of the VQ motif, and the pink vertical line marks the variable residue position inside this motif.
