# Supplementary material for: Genome-wide identification and characterization of VQ genes from cultivated peanut and their response to abiotic stresses
Source: Front Plant Sci. 2026 Jul 3;17:1865834. doi: 10.3389/fpls.2026.1865834 (PMC13376307; doi:10.3389/fpls.2026.1865834)
Supplement: Supplementary Table 3 — Sequence logos for the conserved motifs within VQ proteins. [file Table3.docx]

**Table S3. Sequence logos for the conserved motifs within VQ proteins**

| Motif | E-value | Sites | Width | Logo |
| --- | --- | --- | --- | --- |
| Motif 1 | 1.6e-761 | 71 | 21 | 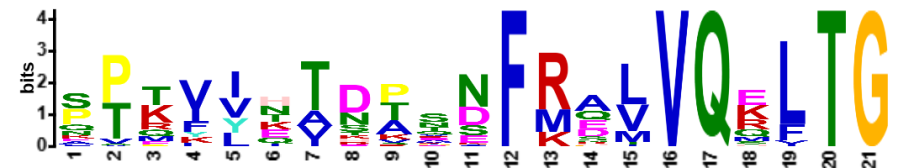 |
| Motif 2 | 3.6e-091 | 71 | 11 | 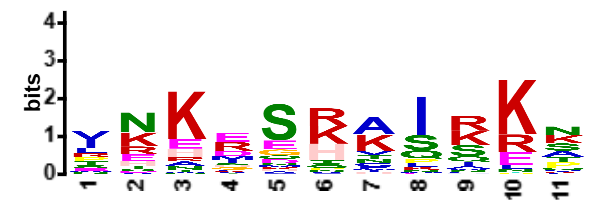 |
| Motif 3 | 4.8e-039 | 71 | 11 | 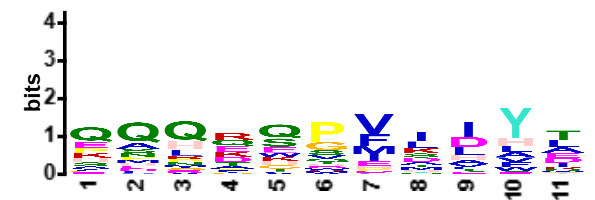 |
| Motif 4 | 3.03e-004 | 71 | 8 | 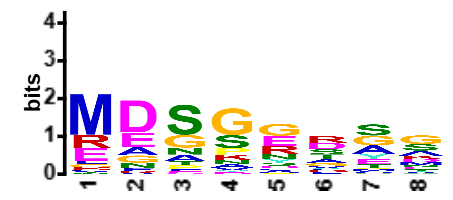 |
| Motif 5 | 1.1e+006 | 71 | 6 | 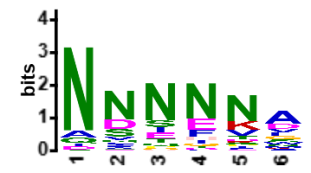 |
| Motif 6 | 1.1e+012 | 71 | 8 | 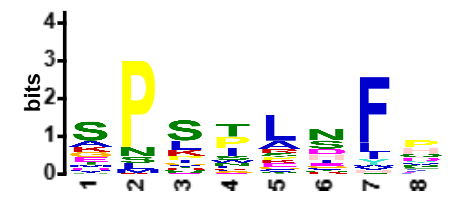 |
| Motif 7 | 1.6e+022 | 71 | 8 | 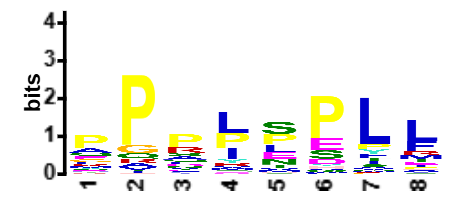 |
| Motif 8 | 5.5e+032 | 71 | 8 | 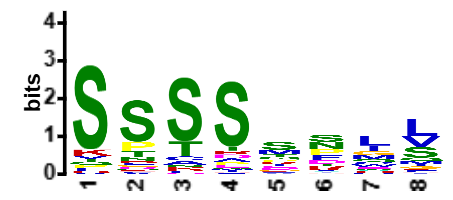 |
| Motif 9 | 7.3e+044 | 71 | 8 | 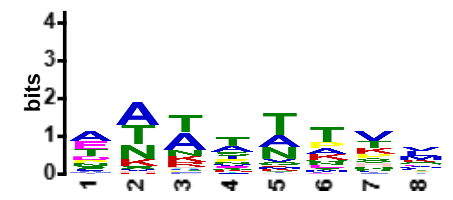 |
| Motif 10 | 5.1e+054 | 71 | 6 | 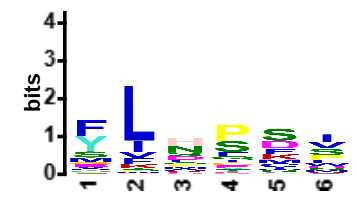 |
